# Effect of a Face-Aging Mobile App–Based Intervention on Skin Cancer Protection Behavior in Secondary Schools in Brazil: A Cluster-Randomized Clinical Trial

**DOI:** 10.1001/jamadermatol.2020.0511

**Published:** 2020-05-06

**Authors:** Titus J. Brinker, Bianca Lisa Faria, Olber Moreira de Faria, Joachim Klode, Dirk Schadendorf, Jochen S. Utikal, Ute Mons, Eva Krieghoff-Henning, Oscar Campos Lisboa, Ana Carla Cruz Oliveira, Henrique Augusto Lino, Breno Bernardes-Souza

**Affiliations:** 1 Department of Dermatology, National Center for Tumor Diseases, German Cancer Research Center, Heidelberg, Germany; 2 School of Medicine, University of Itauna, Itauna, Brazil; 3 Department of Dermatology, Venerology and Allergology, University Hospital Essen, University of Duisburg-Essen, Essen, Germany; 4 Department of Dermatology, Heidelberg University, Mannheim, Germany; 5 Skin Cancer Unit, German Cancer Research Center, Heidelberg, Germany; 6 Cancer Prevention Unit, German Cancer Research Center, Heidelberg, Germany; 7 School of Medicine, Federal University of Ouro Preto, Ouro Preto, Brazil

## Abstract

**Importance:**

Because exposure to UV radiation early in life is an important risk factor for melanoma development, reducing UV exposure in children and adolescents is of paramount importance. New interventions are urgently required.

**Objective:**

To determine the effect of the free face-aging mobile app Sunface on the skin cancer protection behavior of adolescents.

**Design, Setting, and Participants:**

This cluster-randomized clinical trial included a single intervention and a 6-month follow-up from February 1 to November 30, 2018. Randomization was performed on the class level in 52 school classes within 8 public secondary schools (grades 9-12) in Itauna, Southeast Brazil. Data were analyzed from May 1 to October 10, 2019.

**Interventions:**

In a classroom seminar delivered by medical students, adolescents’ selfies were altered by the app to show UV effects on their future faces and were shown in front of their class, accompanied by information about UV protection. Information about relevant parameters was collected via anonymous questionnaires before and 3 and 6 months after the intervention.

**Main Outcomes and Measures:**

The primary end point of the study was the difference in daily sunscreen use at 6 months of follow-up. Secondary end points included the difference in daily sunscreen use at 3 months of follow-up, at least 1 skin self-examination within 6 months, and at least 1 tanning session in the preceding 30 days. All analyses were predefined and based on intention to treat. Cluster effects were taken into account.

**Results:**

Participants included 1573 pupils (812 girls [51.6%] and 761 boys [48.4%]; mean [SD] age, 15.9 [1.3] years) from 52 school classes. Daily sunscreen use increased from 110 of 734 pupils (15.0%) to 139 of 607 (22.9%; *P* < .001) at the 6-month follow-up in the intervention group. The proportion of pupils performing at least 1 skin self-examination in the intervention group rose from 184 of 734 (25.1%) to 300 of 607 (49.4%; *P* < .001). Use of tanning decreased from 138 of 734 pupils (18.8%) to 92 of 607 (15.2%; *P* = .04). No significant changes were observed in the control group. The intervention was more effective for female students (number needed to treat for the primary end point: 8 for girls and 31 for boys).

**Conclusions and Relevance:**

These findings suggest that interventions based on face-aging apps may increase skin cancer protection behavior in Brazilian adolescents. Further studies are required to maximize the effect and to investigate the generalizability of the effects.

**Trial Registration:**

ClinicalTrials.gov Identifier: NCT03178240

## Key Points

**Question:**

Can a face-aging mobile app improve the skin cancer protection behavior of secondary school students?

**Findings:**

In this cluster-randomized clinical trial of 52 school classes with 1573 Brazilian pupils, meaningful improvements were observed in sunscreen use, tanning behavior, and skin self-examinations 3 to 6 months after an intervention using a face-aging app compared with the nonintervention group.

**Meaning:**

Face-aging apps may be useful tools to increase skin cancer protection in adolescents and thereby decrease skin cancer risk.

## Teaser

This randomized clinical trial assesses the effect of the free face-aging mobile app Sunface on the skin cancer protection behavior of adolescents in Brazil.

## Introduction

Melanoma incidence is increasing throughout the world, which results in substantial health and economic burdens.^1,2^ As many as 90% of melanomas are associated with UV exposure, in particular with severe sunburns, and are therefore highly preventable.^1,3,4^ Studies have shown that daily sunscreen use following international dermatology guidelines may prevent sunburns and/or skin cancer, including melanoma.^1,2,5,6^

Brazil has one of the highest UV indexes on earth, and tanning is culturally established. Brazilians frequently experience unprotected overexposure to the sun, especially in childhood and teenaged years.^7^ Citizens in southeastern Brazil are mostly of European descent.^8,9^ Melanoma incidence is high in this area (≤23.5 per 100 000 inhabitants), with little professional screening and an overall skin cancer–specific survival below worldwide rates.^8^

Because the risk of skin cancer is particularly strongly associated with cumulative UV exposure and sunburns early in life,^2,3,6^ several experimental studies aiming at promoting UV protection behaviors among adolescents and young adults have been conducted.^10,11,12,13,14,15^ A school environment provides unique opportunities to propel skin cancer prevention.^16^

Despite the implementation of daily sunscreen use in international dermatologic guidelines, even medical students in Brazil mostly do not use sunscreen and have sunburns at least occasionally.^9^ This lack of exemplary behavior among prospective physicians regarding skin cancer prevention is a known global problem.^17,18^

### Current Knowledge on School-Based Skin Cancer Prevention

Unhealthy behavior regarding UV exposure is mostly adopted in early adolescence, often because a tan is perceived as attractive and future problems, such as melanoma and skin atrophy, seem far away.^14,19,20^ In studies with adolescents and young adults,^12,21,22,23^ appearance-based interventions were more effective than classic health education approaches. An explanation is the strong influence of self-perceived attractiveness on self-esteem in adolescence^24^ because enhancing one’s own attractiveness is a primary motivation for tanning in adolescents.^19^ Appearance-based face-aging interventions have already shown promise in studies in other fields, such as tobacco use prevention.^25,26^ Moreover, a UV-dependent, face-aging desktop program was used in 2 studies that have shown encouraging results but included few patients and had limited applicability to the general population.^21,27^

### The Sunface App

**Figure 1.**
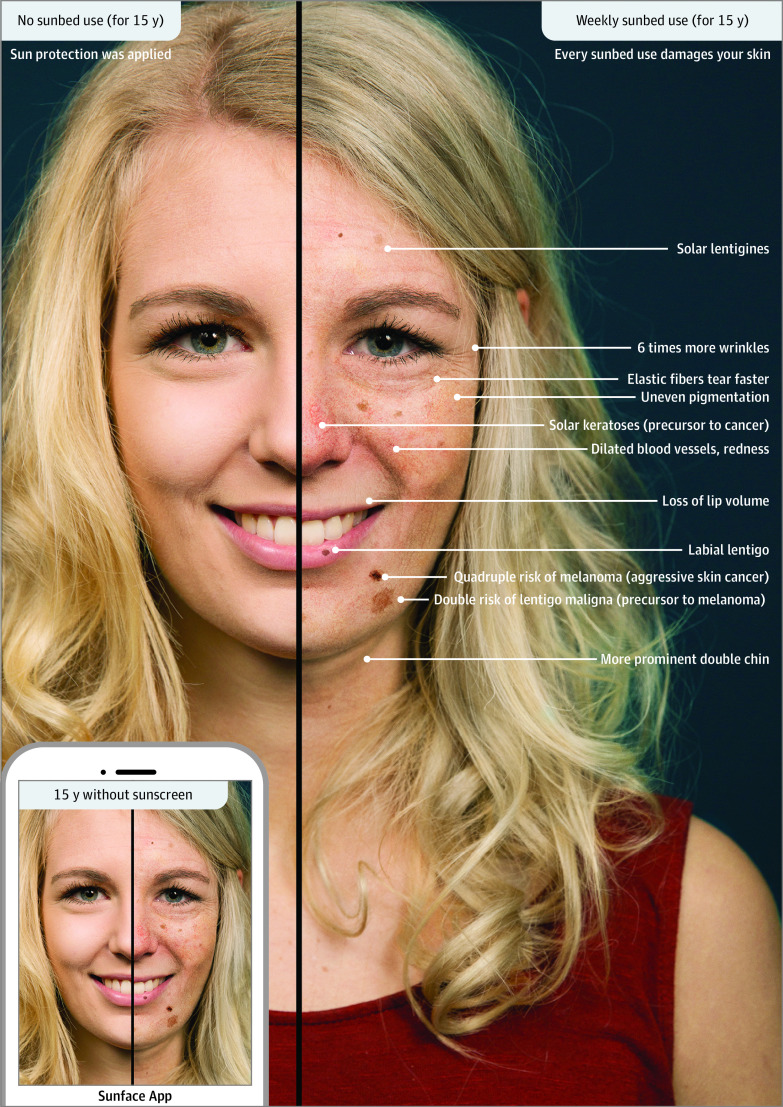
Sunface App The influence of 15 years of weekly sunbed use on the face of a fair-skinned young woman is shown.

We used our freely available mobile phone app, Sunface, which modifies a selfie according to different levels of UV exposure for 5 to 25 years in the future based on individual skin type (see example in Figure 1). Further details about the app were published previously.^23,28,29^ It encompasses the effects of UV on photoaging of the skin in general and on skin cancer development in particular, thus creating photo-aged selfies with sagging skin and spot and wrinkle formation as well as potential malignant skin lesions.

We recently implemented this app in pilot studies in secondary schools via a mirroring approach, wherein the students’ altered 3-dimensional selfies were projected in front of their entire class.^23,28,30^ In a pilot study by Brinker et al^30^ in Brazil, 322 of 356 participants (90.4%) agreed or strongly agreed that this intervention motivated them not to use a tanning bed, and 321 (90.2%) agreed or strongly agreed to increase use of sun protection; only 20 (5.6%) disagreed with both statements. However, effects on actual behavior could not be evaluated in this cross-sectional pilot study.^30^ The present cluster-randomized clinical trial was designed to evaluate whether the implementation of the app in secondary schools is effective in encouraging daily sunscreen use and other skin cancer protection behaviors among adolescents in southeastern Brazil, including potential differences with respect to sex and/or skin type.

## Methods

### Patient Selection and Study Design

The Sunface trial was a randomized clinical superiority trial with 2 parallel groups. Details are found in the peer-reviewed study protocol, which adhered to the Standard Protocol Items: Recommendations for Interventional Trials (SPIRIT) reporting guidelines and was published before we implemented the present trial.^29^ The present study protocol is found in Supplement 1. The study received ethics board approval by the University of Itauna, Itauna, Brazil. All pupils assented to participation, and the ethics board waived the need for written informed consent because no biochemical intervention was involved and data were deidentified.

The study was conducted from February 1 through November 30, 2018. Eight public secondary schools in Itauna were recruited via email, telephone, and personal appointment (in most cases with the principal), and 52 school classes were included in the study.

### Cluster Randomization, Procedure, and Intervention

Within the schools, classes were externally and centrally assigned to control and intervention groups by cluster randomization in a 1:1 ratio of control to intervention participants. Classes were stratified by school grade by a statistician at the University of Duisburg-Essen, Essen, Germany.

For baseline and follow-up surveys, data were collected via written questionnaires. In addition to sociodemographic data (age and sex), the questionnaire captured Fitzpatrick skin type, ancestry of the students, frequency of sunscreen use in the past 30 days, and other skin protection behaviors. The items included in our survey were based on the Sun Exposure and Protection Index questionnaire.^31^ Details can be found in the published study protocol.^29^

The school-based intervention consisting of a 45-minute educational module in the classroom setting using the free face-aging mobile app Sunface and the mirroring approach mentioned previously are described in the published study protocol.^29^ All classes participated in a teacher-supervised baseline survey in February 2018. One week later, the intervention classes received the app-based intervention by centrally trained, local volunteer medical students. Follow-up surveys were conducted 3 and 6 months after the intervention in all classes.

Besides the preintervention (baseline) survey and the 3- and 6-month follow-up surveys, 2 additional surveys were conducted immediately after the intervention. One was designed to evaluate the perception of the intervention by students in the intervention classes, similar to those conducted in the earlier pilot studies.^23,30^ In the second one, the participating medical students filled out a brief process evaluation and motivation questionnaire.

### Outcome Measures and Statistical Analysis

Outcome measures were predefined in our peer-reviewed study protocol and were not changed during implementation or analyses.^29^ The primary end point was change in daily sunscreen use in the 30 days preceding the survey from baseline to 6-month follow-up. Secondary end points included the difference in daily sunscreen use at 3 months of follow-up, at least 1 skin self-examination within 6 months, and at least 1 tanning session in the preceding 30 days. The protocol includes a sample size calculation, which allowed for a loss to follow-up of 40%, predefined methods of data entry, and predefined statistical analyses for the end points.^29^

Data were analyzed from May 1 to October 10, 2019. Statistical analysis was performed using SPSS Statistics, version 24 (IBM Corporation). Intraclass correlation coefficients calculated by the analysis of variance method^32^ for the school level were very low, so that schools were not considered as an additional level in the analyses (eTable 1 in Supplement 2). However, because the intraclass correlation coefficients on the class level were slightly higher, the class was included as an additional random factor in all final models for primary and secondary end points in general mixed models (GENLINMIXED procedure from SPSS) to account for clustering at the class level. This procedure was applied to a binomially distributed dependent variable using a logit link function and an unstructured covariance matrix. The degrees of freedom were adjusted using the Satterthwaite approximation. Robust estimates were used. Sex, Fitzpatrick skin type, and grade at baseline were used for model adjustment. Nevertheless, cluster effects cannot be excluded entirely. Two-sided *P* < .05 indicated significance.

To test the end points, we used special estimated contrast from the models. End points were additionally analyzed according to sex within a common model using special contrasts. All models were developed according to the intention-to-treat principle.

## Results

### Baseline Characteristics of the Study Population

**Figure 2.**
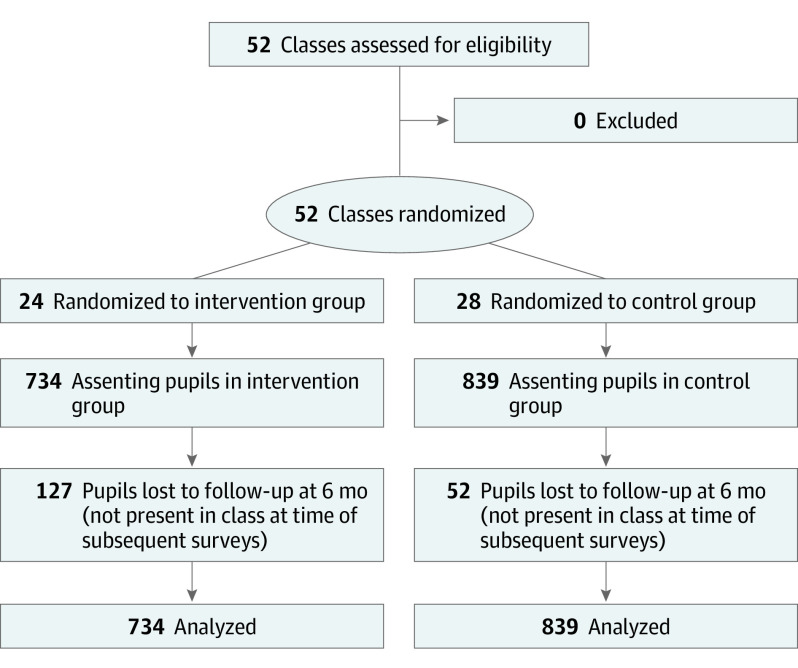
CONSORT Diagram

Participants included 1573 students (812 girls [51.6%] and 761 boys [48.4%]; mean [SD] age, 15.9 [1.3] years) from 52 classes of 8 regular secondary public schools in Itauna. Descriptive characteristics are shown in Table 1, and patient flow is shown in Figure 2. Overall, the relevant characteristics were well balanced between intervention and control groups. Importantly, the use of sunscreen at baseline was very similar in both groups (110 of 734 [15.0%] in the intervention group and 125 of 839 [14.9%] in the control group). The percentage of male students (336 of 734 [45.8%] vs 425 of 839 [50.7%]) and age (mean [SD], 15.7 [1.3] vs 16.0 [1.3] years) were slightly lower in the intervention group. As expected, the proportion of female pupils using sunscreen regularly (169 of 812 [20.8%] vs 66 of 761 [8.7%]), performing skin self-examinations (246 of 812 [30.3%] vs 179 of 761 [23.5%]), and having tanning sessions (171 of 812 [21.1%] vs 76 of 761 [10.0%]) was higher than for male students at baseline (eTable 2 in Supplement 2).

**Table 1.** Descriptive Characteristics of the Participating Students at Baseline

Variable	Student group^a^	
	Intervention	Control
All students	734/1573 (46.7)	839/1573 (53.3)
Classes, No./total No. (%)	24/52 (46.2)	28/52 (53.8)
Sex		
Female	398/734 (54.2)	414/839 (49.3)
Male	336/734 (45.8)	425/839 (50.7)
Age, mean (SD), y	15.7 (1.3)	16.0 (1.3)
School grade		
9th	145/734 (19.8)	113/839 (13.5)
10th	221/734 (30.1)	231/839 (27.5)
11th	178/734 (24.3)	246/839 (29.3)
12th	190/734 (25.9)	249/839 (29.7)
≥1 Parent or grandparent born in Europe	21/734 (2.9)	26/839 (3.1)
Regular use of smartphone	683/734 (93.1)	805/839 (95.9)
Fitzpatrick total sun score, mean (SD)^b^	19.2 (4.0)	18.8 (4.3)
Fitzpatrick skin type		
I or II	46/734 (6.3)	70/839 (8.3)
III	256/734 (34.9)	293/839 (34.9)
IV	368/734 (50.1)	421/839 (50.2)
V	64/734 (8.7)	55/839 (6.6)
≥1 Skin self-examination in the preceding 6 mo	184/734 (25.1)	241/839 (28.7)
Female	109/398 (27.4)	137/414 (33.1)
Male	75/336 (22.3)	104/425 (24.5)
≥1 Tanning session in the preceding 30 d	138/734 (18.8)	109/839 (13.0)
Female	97/398 (24.4)	74/414 (17.9)
Male	41/336 (12.2)	35/425 (8.2)
Daily sunscreen use in the preceding 30 d	110/734 (15.0)	125/839 (14.9)
Female	79/398 (19.8)	90/414 (21.7)
Male	31/336 (9.2)	35/425 (8.2)

^a^ Unless otherwise indicated, data are expressed as number/total number (percentage) of students.

^b^ Scores range from 0 (high sensitivity) to 32 (robust).

### Perception of the Intervention

Immediately after the intervention, 690 of 734 pupils (94.0%) rated the intervention as fun and informative and claimed that it motivated them to use sunscreen (662 of 734 [90.2%]) and perform skin self-examination (668 of 734 [91.0%]) (eTable 3 in Supplement 2). In all classes, the medical students agreed or fully agreed that they had had an empathic communication with the adolescents, that the intervention was enjoyable, and that their participation motivated them to discuss sun protection with their future patients. For the process evaluation, the medical students indicated that the intervention was conducted as outlined in the protocol in all intervention classes.

### Primary End Point: Daily Sunscreen Use at 6-Month Follow-up

**Figure 3.**
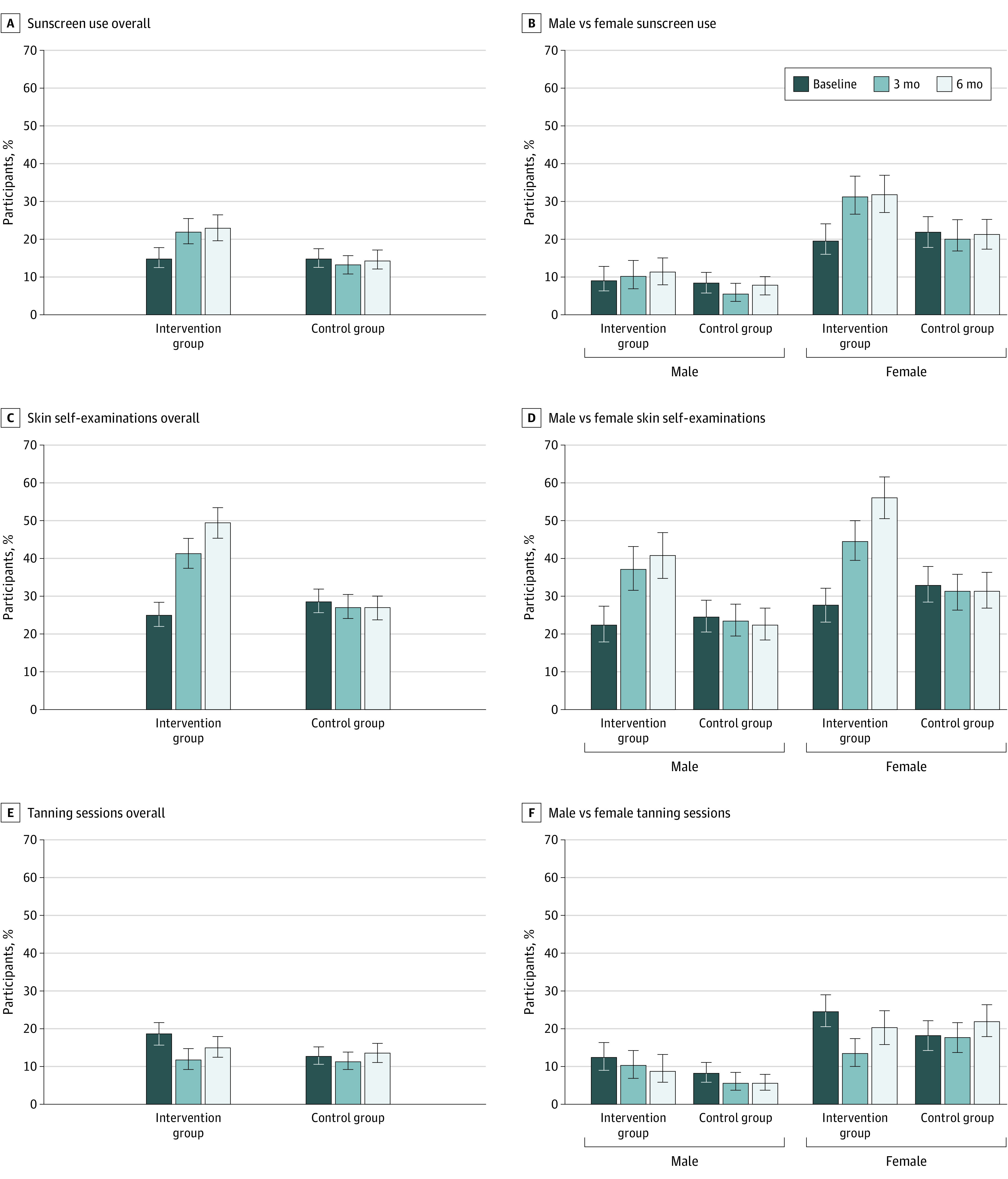
Sunscreen Use, Skin Self-examinations, and Tanning Sessions of Student Participants The diagrams show the percentage of pupils for every end point at baseline and 3 and 6 months after the intervention. Error bars represent Clopper and Pearson 95% CIs. Underlying data are shown in eTable 4 in Supplement 2 for parts A and B; eTable 11 in Supplement 2 for parts C and D; and eTable 15 in Supplement 2 for parts E and F.

Whereas 110 of 734 students in the intervention group (15.0%) used sunscreen daily at baseline, this increased to 139 of 607 (22.9%) 6 months after the intervention. In the control group, in contrast, this percentage did not increase (14.9% [125 of 839] at baseline to 14.5% [114 of 787] at 6 months) (Figure 3A and eTable 4 in Supplement 2). The difference in change between intervention and control groups was 8.2% in favor of the intervention group (95% CI, 4.2%-12.2%; *P* < .001) (eTable 5 in Supplement 2).

Because there were major differences in UV protection behavior between the sexes at baseline, we also performed separate analyses for female and male students. Daily sunscreen use among female students in the intervention group was 79 of 398 (19.8%) at baseline and 108 of 338 (32.0%) at 6 months. According to our model, for female students, the difference in change between intervention and control groups was 12.8% in favor of the intervention (95% CI, 6.2%-19.3%; *P* < .001). Empirically, a slight increase was also observed among male students, with 31 of 336 (9.2%) regularly using sunscreen at baseline and 31 of 269 (11.5%) at 6 months. The difference in change between intervention and control groups among male students calculated with the model was 3.3% in favor of the intervention. However, this difference was not statistically significant (95% CI, –1.6% to 8.3%; *P* = .19) (eTable 6 in Supplement 2).

Patterns of sunscreen use, overall and by sex, at baseline and 3 and 6 months are depicted in Figure 3A-B. Fitzpatrick skin type had a small effect on the proportion of pupils who used sunscreen daily, although baseline levels were higher in pupils with very light or light skin (intervention group, 11 of 46 [23.9%]; control group, 18 of 70 [25.7%]) (eTables 4 and 7 in Supplement 2).

### Secondary End Point: Daily Sunscreen Use at 3-Month Follow-up

The results obtained for the 3-month follow-up (sunscreen use, 136 of 618 [22.0%] in the intervention group) were very similar to those obtained for the 6-month follow-up (139 of 607 [22.9%]) (Figure 3A-B; see eTable 8 in Supplement 2 for influencing factors, eTable 9 in Supplement 2 for overall differences, and eTable 10 in Supplement 2 for sex-specific difference in change). These findings indicate that most of the students took up daily sunscreen use early after the intervention and maintained this behavior for a longer period.

### Secondary End Point: Skin Self-examinations

Regarding skin self-examinations, the study also showed significant improvements in the intervention group relative to the control group (Figure 3C-D and eTable 11 in Supplement 2). In the intervention group, 184 of 734 pupils (25.1%) had performed at least 1 skin self-examination within the past 6 months at baseline, whereas 300 of 607 (49.4%) had done so at 6 months after the intervention. In the control group, 241 of 839 pupils (28.7%) had performed at least 1 skin self-examination at baseline and 211 of 787 (26.8%) at the 6-month follow-up. The difference in change between the intervention and control groups was 26.4% in favor of the intervention (95% CI, 21.1%-31.6%; *P* < .001) (eTable 12 in Supplement 2). For female students, the relative difference was 30.0% (95% CI, 22.7%-27.3%; *P* < .001); for male students, it was 21.5% (95% CI, 14.1%- 28.9%; *P* < .001) (eTable 13 in Supplement 2). The proportion of pupils who performed at least 1 skin self-examination did not differ significantly by Fitzpatrick skin type (eTables 11 and 14 in Supplement 2).

### Secondary End Point: Tanning Sessions

Overall, 138 of 734 pupils (18.8%) in the intervention group had had a tanning session within the past 30 days at baseline. Six months after the intervention, that number decreased slightly to 92 of 607 (15.2%). In the control group, 109 of 838 students (13.0%) had tanning sessions at baseline, and that number did not decrease after 6 months (107 of 787 [13.6%]) (Figure 3E and eTable 15 in Supplement 2). The calculated relative overall difference in change to baseline values was −4.6% (95% CI, −8.3% to –0.9%; *P* = .02) 3 months after the intervention and −4.1% (95% CI, −8.0 to −0.2%; *P* = .04) 6 months after the intervention (eTable 16 in Supplement 2). Thus, a decrease in tanning sessions observed after the 3-month follow-up was only partially maintained during the following 3 months.

Again, results differed by sex (Figure 3F and eTable 17 in Supplement 2). Whereas the number of female students who had tanning sessions in the intervention group decreased from 97 of 398 (24.4%) to 46 of 343 (13.4%) by 3 months after the intervention, this number increased almost to baseline values after 6 months (68 of 338 [20.1%]). Nevertheless, the calculated difference in change in tanning sessions observed 3 months after the intervention for the female students (10.4%; 95% CI, −16.7% to −4.1%; *P* = .001) had not disappeared entirely after 6 months (8.0%; 95% CI, −14.5% to −1.5%; *P* = .02). For the male students, no significant effects of the intervention on the number of tanning sessions were detectable (difference at 6 months, 0.8%; 95% CI, −5.6% to 4.0%; *P* = .75). Moreover, the amount of tanning among male students was lower than that among female students at baseline (intervention group, 41 of 336 [12.2%] vs 97 of 398 [24.4%]; control group, 35 of 425 [8.2%] vs 74 of 414 [17.9%]). No significant differences in adolescents’ tanning behavior were noted by Fitzpatrick skin type (eTables 15 and 18 in Supplement 2).

### Effectiveness of the Intervention

To assess the effectiveness of the face-aging intervention regarding the different end points, we also calculated numbers needed to treat (Table 2) for the 3 different end points based on absolute risk reductions calculated with the model at 6 months after the intervention. The results confirm the higher effectiveness of the intervention in female students (number needed to treat for the primary end point: 8 for girls and 31 for boys).

**Table 2.** Numbers Needed to Treat (NNT) According to the Observed Absolute Risk Reduction at 6-Month Follow-up

End point	NNT (95% CI)^a^
Daily sunscreen use	
Overall	13 (9-25)
Female	8 (6-17)
Male	31 (13-∞)^b^
Skin self-examinations	
Overall	4 (4-5)
Female	4 (3-5)
Male	5 (4-8)
Tanning sessions	
Overall	25 (13-500)
Female	13 (7-67)
Male	125 (18-∞)^b^

^a^ Calculated as 1 divided by the observed absolute risk reduction.

^b^ Difference in change not significant.

### Attrition Analysis

Of 1573 participants at baseline, 179 (11.4%) had dropped out at the 6-month follow-up. Thus, the attrition rate was much lower than our predefined maximum of 40%. However, a comparison between study arms showed that the dropout rate was almost 3 times as high in the intervention group (127 of 734 [17.3%] vs 52 of 839 [6.2%]) (eTable 19 in Supplement 2). We found no difference in attrition rates between intervention and control groups with respect to daily sunscreen use, skin self-examinations, or tanning behavior (eTables 20-22 in Supplement 2), suggesting a low potential for attrition bias concerning the primary and secondary end points. However, the attrition rates were higher among male students (100 of 761 [13.1%]) compared with female students (79 of 812 [9.7%]) (eTable 23 in Supplement 2).

## Discussion

To our knowledge, this is the first school-based cluster-randomized clinical trial on skin cancer prevention using a face-aging mobile app. The trial was built on previous studies suggesting that appearance-based approaches are more effective in changing the behavior of adolescents and young adults than traditional health education approaches.^12,21,22,23,25,26,28^ Using a face-aging mobile app in skin cancer prevention is a novel strategy that makes use of the current technological options and takes widely accepted theories for behavioral change into account.^29^

The intervention used in this study was effective in convincing a substantial part of the students to take up regular sunscreen use and to examine their own skin regularly. Moreover, these effects were maintained for at least half a year. In contrast, the effect on tanning sessions appeared less sustainable: we observed a clear decrease in the proportion of female students who tanned at the 3-month follow-up, but this effect was partially lost during the subsequent months. This discrepancy may be explained by the fact that often adopting a new healthy behavior, such as sunscreen use, is easier than completely shedding a (bad) habit, such as tanning, especially if that habit is associated with a short-term benefit, such as greater immediate attractiveness. Indeed, tanning has been described as an addictive behavior.^14^ A possible way to address this phenomenon might be to repeat the intervention regularly or to alternate this intervention with other interventions also aimed at improving sun protection behaviors. Starting the intervention earlier in life before behavioral patterns are fixed might also help to increase the benefit of the intervention regarding tanning sessions.

In a modeling study based on previous studies, Olsen et al^33^ calculated the potential effects of a school-aged UV protection intervention. They concluded that cumulative melanoma incidence in the 70 years after the intervention would decrease by approximately 20% if all children started using sunscreen regularly. Thus, the effects we have observed in this study could contribute significantly to the reduction of skin cancer rates, especially if the intervention was repeated and combined with other strategies aimed at promoting skin cancer awareness and UV protection.

### Limitations

The study was conducted in Brazil only, potentially limiting its generalizability. Also, the effects were mostly driven by the female students. With respect to the future implementation of the Sunface app for skin cancer prevention, the fact that almost 3 times as many students from the intervention group dropped out of the study has to be considered as a potential source of bias. The study showed a clear overall improvement in UV protection behavior, and only a small minority of participants indicated negative perceptions toward the intervention. Nevertheless, the intervention may have led to strong adverse reactions in some students, leading to the observed higher dropout rate in the intervention group. Seeing one’s own face unfavorably altered by the app, especially when projected in front of the entire class, may have triggered attempts to avoid further confrontation with the subject. Teasing by other pupils might have reinforced such an unwanted effect.

Adverse reactions to the intervention might be avoided by omitting the mirroring altogether or by only mirroring the altered selfies of the medical students performing the intervention and/or by moderating the changes seen in the app. However, this approach could weaken the desired effects of the intervention on other students, implying that potential alterations will have to be considered and tested carefully.

## Conclusions

We observed improvements during a 6-month period in skin cancer protection behavior among the participating students in response to the intervention. We therefore consider the face-aging, app-based skin cancer prevention strategy promising and plan to pursue it in further studies. Further research should be dedicated to investigating how to best implement interventions such as the present one into public health systems to maximize their effect on skin cancer prevention, with a particular focus on increasing effectiveness for male students.

## Notes

Supplement 1. Trial Protocol

Supplement 2. eTable 1. Intraclass Correlation Coefficients (ICCs) Determined by the ANOVA Method eTable 2. Bivariate Relations at Baseline (All Cases Including Dropouts) eTable 3. Perception of Students on the Intervention–Immediate Postintervention Survey eTable 4. Descriptive Characteristics of Pupils With Daily Use of Sunscreen During the Past 30 Days at Baseline and 3- and 6-Month Follow-up eTable 5. Comparison of the Change of Daily Sunscreen Use Between the Intervention and Control Groups eTable 6. Gender-Specific Comparison of the Change of Daily Sunscreen Use Between the Intervention and Control Groups eTable 7. Analysis of the Influence of Different Factors on the Primary End Point eTable 8. Analysis of the Influence of Different Factors on the Secondary End Point “Daily Sunscreen Use at 3-Month Follow-up” eTable 9. Comparison of the Change of Daily Sunscreen Use Between the Intervention and Control Groups eTable 10. Gender-Specific Comparison of the Change of Daily Sunscreen Use Between the Intervention and Control Groups eTable 11. Descriptive Characteristic of Pupils With at Least 1 Skin Self-examination in the Past 6 Months at Baseline and 3- and 6-Month Follow-up eTable 12. Comparison of the Change of the Prevalence of Pupils With at Least 1 Skin Self-examination Between the Intervention and Control Groups eTable 13. Gender-Specific Comparison of the Change of the Prevalence of Pupils With at Least 1 Skin Self-examination Between the Intervention and Control Groups eTable 14. Analysis of the Influence of Different Factors on the Secondary End Point “Skin Self-examination Within the Past 6 Months” eTable 15. Descriptive Characteristic of Pupils With at Least 1 Tanning Session in the Past 30 Days at Baseline and 3- and 6-Month Follow-up eTable 16. Comparison of the Change of the Prevalence of Pupils With at Least 1 Tanning Session in the Past 30 Days Between the Intervention and Control Groups eTable 17. Gender-Specific Comparison of the Change of the Prevalence of Pupils With at Least 1 Tanning Session in the Past 30 Days Between the Intervention and Control Groups eTable 18. Analysis of the Influence of Different Factors on the Secondary End Point “at Least 1 Tanning Session Within the Past 30 Days” eTable 19. Dropouts in Relation to Assigned Group eTable 20. Dropouts in Relation to Sunscreen Use eTable 21. Dropouts in Relation to Skin Self-examinations eTable 22. Dropouts in Relation to Tanning Sessions eTable 23. Dropouts in Relation to Gender Supplement 3. Data Sharing Statement
