# Supplementary material for: Effect of a Face-Aging Mobile App–Based Intervention on Skin Cancer Protection Behavior in Secondary Schools in Brazil: A Cluster-Randomized Clinical Trial
Source: JAMA Dermatol. 2020 May 6;156(7):737–45. doi: 10.1001/jamadermatol.2020.0511 (PMC7203674; doi:10.1001/jamadermatol.2020.0511)
Supplement: Supplement 1. — Trial Protocol [file jamadermatol-156-737-s001.pdf]

UNIVERSITY OF ITAUNA – UIT

Research Project

Project title: Effectiveness of a medical student-delivered photoaging intervention for skin cancer prevention in Brazilian secondary schools: randomized controlled study

Research Project to be submitted to the Research Ethics Committee of the University of Itauna

Responsible investigator: Prof. Dr. Olber Moreira de Faria[1]

[1]Associate Professor of Medicine at UIT School of Medicine

Itauna, MG, Brazil

October, 2017

ABSTRACT

The incidence of melanoma is increasing faster than any other major cancer both in Brazil and worldwide. The southeast of Brazil has especially high incidences of melanoma, and early detection is low. Exposure to UV radiation represents a primary risk factor for developing melanoma. Increasing attractiveness is a major motivation for adolescents for tanning. A medical student-delivered intervention that harnesses the broad availability of mobile phones as well as adolescents' interest in their appearance may represent a novel method to improve skin cancer prevention. We developed a free mobile app ("Sunface"), which will be implemented in at least 30 secondary school classes, each with 21 students (at least 30 classes with 21 students for control) in February 2018 in the city of Itauna (Brazil) via a novel method called mirroring. In a 45-minute classroom seminar, the students' altered three-dimensional selfies on tablets are "mirrored" via a projector in front of their entire class, showing the effects of unprotected UV exposure on their future faces. External block randomization via computer is performed on the class level with a 1:1 allocation. Sociodemographic data, as well as skin type, ancestry, UV protection behavior and its predictors are measured via a paper-pencil questionnaire before as well as three and six months post-intervention. The primary endpoint is the group difference in the 30-day prevalence of daily sunscreen use at a six-month follow-up. Secondary endpoints include (1) the difference in daily sunscreen use at a three-month follow-up, (2) if a self-skin exam in accordance with the ABCDE rule was performed within the six-month follow-up and (3) the number of tanning sessions.

INDEX

Introduction …………………………………………………………………………. page 04

Current knowledge on school-based skin cancer prevention …………. page 05

Introduction of the Sunface app ……………………………………….... page 06

Methods and Analysis ……………………………………………………….... page 12

Study design ………………………………………………………….….. page 12

Intervention …...…………………………………………………….…. page 13

Participants …...……………………………………………………….…. page 15

Eligibility criteria at baseline ……………………….. page 15

Contaminated classes ……………………………… page 15

Procedure ……………………………………………………………….... page 15

Data collection …………………………………………………...…. page 16

Cluster randomization ………………………………………………... page 16

Outcomes …………………………………………………………….... page 16

Statistical considerations ……………………………..…………….... page 17

Sample size calculation ……………………………. page 17

Data entry ……………………………………………. page 17

Analysis ………………………………………….…... page 17

Discussion ………………………………………………………….……….. page 18

Limitations ……………………………………………………………... page 19

Ethics and Dissemination ……………..…………………………………….... page 20

Conflicts of Interest ………………..…………………………………….…... page 20

Funding ……………..…………………………………………………….…... page 21

References ……………...……….………………………………………….. page 21

Appendix – Questionnaire ……………..……………………………….…. page 26

Introduction

According to the World Health Organization, the incidence of melanoma is increasing more rapidly than any other major cancer both in Brazil and worldwide. Melanoma is one of the most common cancers in young adults and poses substantial health and economic burdens [1].

Approximately 90% of melanomas are associated with UV exposure, in particular with the frequency of severe sunburns, and are therefore highly preventable [2]. Multiple studies showed that daily sunscreen use with a sun protection factor (SPF) above 30, as recommended by international dermatology guidelines, may prevent sunburns and skin cancer including melanoma [3-6].

Brazil has one of the highest UV indexes on earth; additionally, tanning is culturally established and Brazilians commonly experience unprotected overexposure to the sun, especially in their childhood and teenage years [7-11]. In a 2008 population-based survey with 1,604 participants in the south of Brazil, 48.7% reported at least one sunburn in the prior year [10]. In an attempt to mitigate the health damage caused by excessive UV exposure, Brazil was the first country to prohibit indoor tanning in 2009, albeit with limited success [9]. The southeast of Brazil (the location of this study) is especially populated by citizens with a European ancestry and therefore has high incidences of melanoma (up to 23.5/100,000 inhabitants) with a lack of early diagnosis and an overall survival below worldwide rates [12-15].

Interventions encouraging sun protection habits are important, particularly among adolescents, as increased risk of skin cancer is associated with cumulative UV exposure and sunburns early in life [16-18]. In line with this association, various recent experimental studies to test these effects in young target groups aimed at

promoting sunscreen use as an end point [19-22], and others used various UV protection behaviors (including avoiding sunbeds) or behavior scores [23-32]. Given the substantial amount of time that children and adolescents spend in the school environment, addressing skin cancer prevention in this setting is crucial and provides a unique opportunity to propel skin cancer prevention programs [33].

Despite the effectiveness of daily sunscreen and its implementation in international dermatologic guidelines [34], a study conducted in Brazil among 398 medical students from the city of Curitiba showed that only 8.4% use sunscreen daily, 4.3% had already used tanning beds and 85.5% had past sunburns despite having undergone a clinical rotation in a Dermatology department [8]. The lack of exemplary behavior among prospective physicians regarding skin cancer prevention is known on a global scale [35-37]. The authors of this study have concluded that novel engagement methods are needed to answer the increasing demand for skin cancer awareness among physicians.

The Sunface mirroring intervention aims to provide science-based skin cancer prevention to a large number of adolescents in an attempt to sensitize prospective physicians to the importance of exemplary behavior [38, 39].

Current knowledge on school-based skin cancer prevention

Unhealthy behavior with respect to UV exposure is mostly initiated in early adolescence [40], commonly with the belief that a tan increases attractiveness [41-43] and the problems related to melanoma as well as skin atrophy are too far in the future to fathom.

A recent randomized trial with Australian high school students demonstrated that appearance-based videos on UV-induced premature aging were superior in encouraging sunscreen use to videos of the same length focusing exclusively on health aspects [19]. These findings are in line with international studies demonstrating the important influence of self-perceived attractiveness on self-esteem in adolescence [44, 45]. Furthermore, enhancing one's attractiveness is a primary motivation for tanning in adolescents both in Brazil and worldwide [41, 46, 47]. In addition, the success of appearance-based photoaging intervention mobile apps, in which an image is altered to predict future appearance in the fields of tobacco and adiposity prevention, shows promise for these interventions in behavioral change settings [48-53].

In the setting of skin cancer prevention, a quasi-experimental study by Williams et al. demonstrated significantly higher scores for predictors of sun protection behavior in young women from the UK (70 participants in total) using a photoaging desktop program [54]. Furthermore, the photoaging software 'showed promising reduction in young adults' tanning intentions in a study with ten participants in total (7 female and 3 male) [55]. However, prior studies are limited by their small sample size and limitations related to expanding the target population.

Introduction of the Sunface app

We harnessed the widespread availability of mobile phones and adolescents' interest in appearance to develop the free mobile phone app "Sunface," which enables the user take a selfie and then offers three categories: "daily sun protection", "no sun protection" and "weekly tanning," showing the altered face at 5 to 25 years in the future (Figure 1, Figure 2, Figure 3, Figure 4). All effects are based

on the individual skin type that the user can choose at the start of the app (Figure 5). The app also shows the most common UV-induced skin cancers via extra buttons and calculates how the odds ratio is increased with different behaviors. In addition, the app gives advice on sun protection, explains the facial changes and encourages skin examinations using the ABCDE rule (assess border irregularity, color variety, diameter, and evolution [56]).

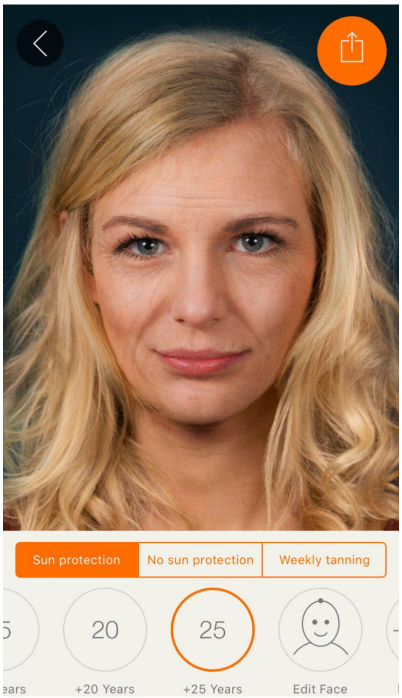

Figure 1: Effect view: 25 years of skin ageing with sun protection.

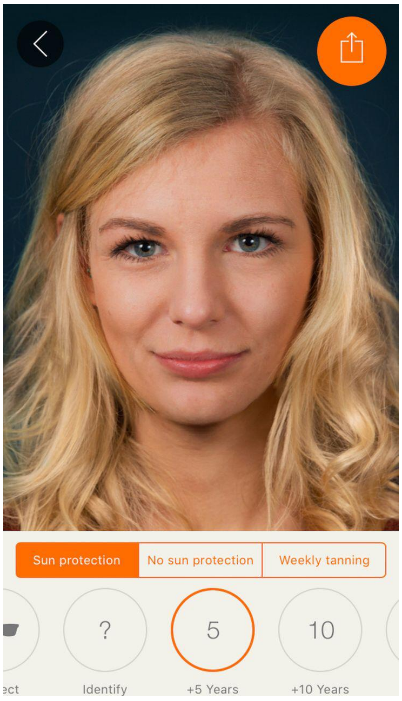

Figure 2: Effect view: 5 years of skin ageing with sun protection.

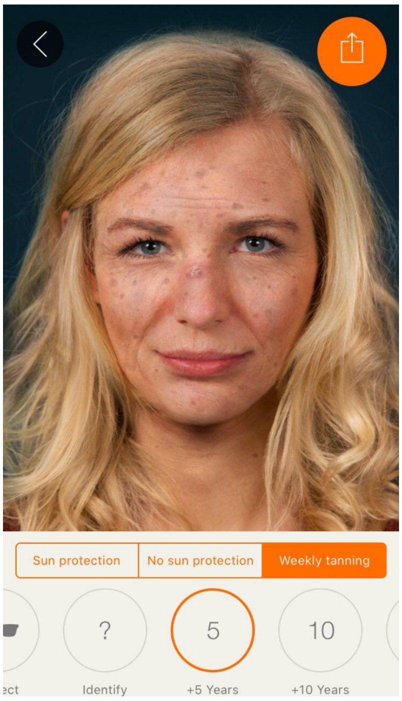

Figure 3: Effect view: 5 years of weekly tanning without sun protection.

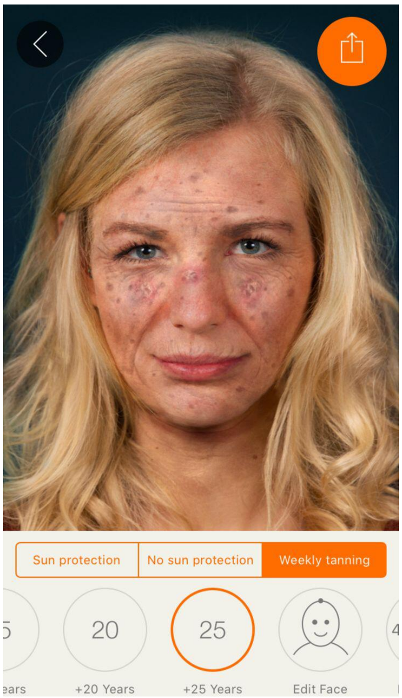

Figure 4: Maximum effect view: 25 years of UV damage due to weekly tanning.

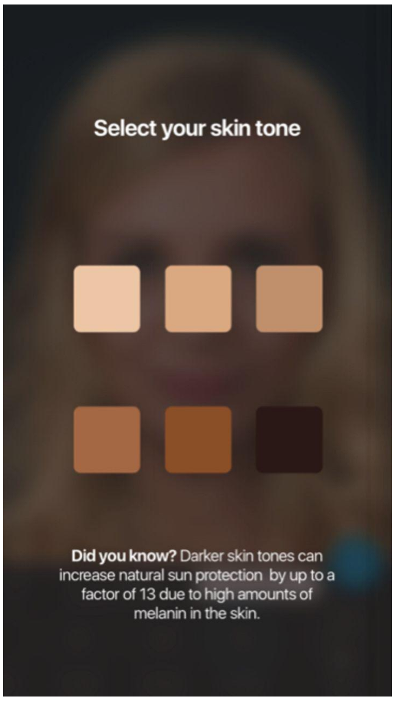

Figure 5: Start screen of the app prompts the user to pick his skin type.

Afterwards, the app offers many sharing options (animated video or photo) with family and friends. By this means, the social network of the user may also be informed about the various photoaging effects of excessive UV exposure and potential health consequences, as well as potentially learning about the benefits of using the app [57].

To produce realistic effects (Figure 6) and to show the user realistic odds ratios for the options they choose in the app for the three most strongly associated skin pathologies, an extensive review of the current literature on UV-induced skin damage [58, 59] was conducted for each specific skin type. As no trials with 25 years of follow-up were available, we had to extrapolate the current evidence on UV-induced skin damage for the specific skin types. The evidence consists of more than 50 publications to create realistic effects from a clinician’s standpoint (which may differ from what the average person perceives as realistic).

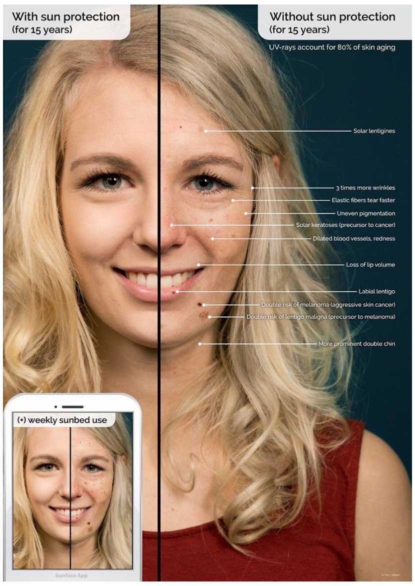

Figure 6: Explanatory graphic of the effects within the app.

We recently implemented this app in 2 German secondary schools via a method called mirroring. We “mirrored” the students’ altered 3-dimensional (3D) selfies on mobile phones or tablets via a projector in front of their entire grade. Using an anonymous questionnaire, we then measured sociodemographic data as well as risk factors for melanoma and the perceptions of the intervention on a 5-point Likert scale among 205 students of both sexes aged 13-19 years (median 15 years).

In our pilot study, we found more than 60% agreement in both items measuring motivation to reduce UV exposure and only 12.5% disagreement: 126 (63.0%) agreed or strongly agreed that their 3D selfie motivated them to avoid using a tanning bed, and 124 (61.7%) agreed or strongly agreed to increase use of sun protection; only 25 (12.5%) disagreed with both items. However, no effects on actual behavior could be measured due to the cross-sectional design of the study [60].

This randomized trial was designed to answer the following questions:

Is the implementation of the app in secondary schools in southeastern Brazil effective in encouraging daily sunscreen use among adolescents? Is it equally effective for both genders? Is it effective for the most sensitive skin types? How does the app intervention change the attitudes towards sun protection in accordance with the theory of planned behavior [61]?

Methods and Analysis

Study Design

The Sunface trial is designed as a randomized controlled superiority trial with two parallel groups. Our primary end point is the difference between the two groups in daily sunscreen use (past 30 days) from baseline to six months follow-up (Figure 7). The planned study period is February 2018 to November 2018. The study groups will consist of randomized classes receiving the intervention and control classes within the same schools (no intervention). Randomization is externally and centrally performed at the school level with a 1:1 allocation (control:intervention) via computer [12]. A total of at least 60 secondary school classes in Itauna, Brazil will participate in the teacher-supervised baseline survey in February 2018, which is conducted by trained data collectors. One week after the baseline survey, the intervention classes receive a 45-minute app-based intervention conducted by local medical student volunteers. Follow-up surveys will be conducted 3 and 6 months post-intervention.

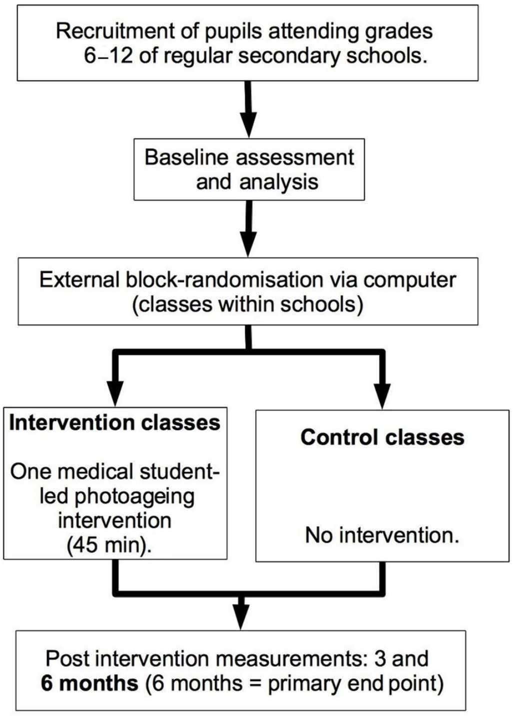

Figure 7: Study design.

Intervention

The school-based intervention under evaluation consists of a 45-minute educational module in the classroom setting using a photoaging app. The intervention is presented by 2 medical students per classroom to approximately 21 students at a time. The goal is to initiate and guide the student evaluation process of skin cancer prevention with age-appropriate information that helps the students reframe positive opinions and views regarding sun protection habits in a gain-framed and interactive manner.

To integrate app-based photoaging interventions into a school-based setting, we previously developed and tested the mirroring approach in a pilot study [49]. Mirroring means that the students' altered three-dimensional selfies on smartphones or tablets are "mirrored" via a projector in front of the entire class. The mirroring approach is implemented by medical student volunteers from the University of Itauna, who receive standardized training in advance. To ensure the participation of all students within a certain class and to avoid contamination within schools, we will implement the mirroring intervention via 10 Samsung Galaxy Tablets that are already set up and brought to the schools by the volunteers.

In the first 10-minute phase, the displayed face of one student volunteer is used to show the app's altered features in the three categories to the peer group, providing an incentive for the rest of the class to test the app. Students can interact with their own animated face via touch. In front of their peers, they will be able to display their image as a non-/sun protection user/weekly tanning bed user at 5, 10, 15, 20 and 25 years in the future (see Figures 1-4). Multiple device displays can be projected simultaneously, which are used to consolidate the altered measures with graphics (e.g., to explain wrinkle formation). We implement mirroring with Galaxy Tab A

(Samsung) via Apple’s proprietary AirPlay interface using the Android app “Mirroring360” (Splashtop Inc.).

In the second 15-minute phase, students are encouraged to try the app on one of the tablet computers. The number of provided tablet computers was calculated so that the phase would take up to 12 minutes at the most after factoring in a utilization time of approximately 4 minutes per student. By this calculation, 25 minutes of the mirroring intervention and 10 provided tablets were sufficient to have every student within a class of 40 pupils successfully photoaged at least once.
In the following 15 minutes, the remaining functions of the app are discussed with the students: facial changes, the ABCDE rule and the guidelines for sun protection are addressed in an interactive setting. At the end of the classroom seminar, we ask for the students' final judgments on daily sunscreen use to create positive peer pressure and influence the students’ subjective norm in accordance with the theory of planned behavior [61].

In the last 5 minutes, the perception of the intervention by the students is measured directly after the intervention in an anonymous survey on a 5-point Likert scale via four items: (1) “The animation of my 3D selfie motivates me to use daily sunscreen,” (2) “I learned new benefits of sun protection,” (3) "The intervention motivates me to check my skin with the ABCDE rule in the next six months," and (4) “The intervention was fun.”

Participants

Eligibility criteria at baseline

Students from Itauna in southeast Brazil attending grades 6 to 12 in all types of regular secondary school are eligible.

Contaminated classes

All classes will be included in the final intention-to-treat analysis. However, app use will be assessed in both groups at six months follow-up to assess contamination of control classes and will be the basis for a secondary (sensitivity) analysis with the methods described in the Analysis section of this protocol.

Procedure

The schools are recruited via E-mail, telephone and personal appointment (in most cases with the principal). Reasons for non-participation are not recorded. Data are collected via a paper-pencil questionnaire. In addition to sociodemographic data (age, gender and school type), the questionnaire captures the Fitzpatrick skin type, the ancestry of the school students [62] and the frequency of sunscreen use in the past 30 days as well as other sun protection behaviors. These items are based on the Sun Exposure and Protection Index questionnaire [63] and were either used in their original form or adapted to the specific circumstances of the present study (the final questionnaire is included as an Appendix in the end of this file). No Portuguese equivalents of the instruments were available; thus, we used the Conceptual Method for translation described by the WHO/UNESCAP Project on Health and Disability Statistics [64]. Newly translated and/or modified items were extensively pretested and subjected to statistical analyses (internal consistency/Cronbach's α and

exploratory and confirmatory factor analyses, which represented the basis for item selection).

Data Collection

Each data collector received training for data collection and was required to use an adapted standardized protocol for data collection, an optimized version of that used in the Smokerface randomized trial [65].

Cluster randomization

In accordance with the guidelines for good epidemiologic practice, classes within schools are externally and centrally randomly assigned to the control or intervention group via block randomization in a 1:1 ratio (control:intervention) via computer by a statistician at the University of Duisburg-Essen, Germany. Stratification will be performed by grade.

Outcomes

The primary endpoint is the difference in the 30-day-prevalence of daily sunscreen use between both groups at six months follow-up. Secondary endpoints include the difference in daily sunscreen use at three months follow-up if a self-skin exam in accordance with the ABCDE rule [56] was performed within the six-month follow-up and the number of tanning sessions in the past 30 days. For all end points, the number needed to treat will also be calculated. A daily sunscreen user is defined as a pupil who claims to have used sunscreen daily or almost daily in the 30 days preceding the survey.

Statistical considerations

Sample size calculation

We calculated sample sizes of 630 in the intervention group and 630 in the control group, which were obtained by sampling 30 classes with 21 students each in the intervention group and 30 classes with 21 students each in the control group to achieve 80% power to detect a prevalence difference between the groups of 5%. The daily sunscreen prevalence was assumed to be 6% under the null hypothesis and 11% under the alternative hypothesis based on a small pilot survey with 150 students in Itauna. The test statistic used is the two-sided score test (Farrington & Manning). The significance level of the test is 0.05. Normal class size in Brazil is 35 pupils; a lost to follow-up effect of 40% was taken into account.

Data entry

Data entry will be supported by the current software version of Formic Fusion by Xerox AG (Kloten, Switzerland) and the recommended scanners.

Analysis

To examine baseline differences in pupils' characteristics in our experimental design, we will use χ^2 tests for categorical variables and t-tests for continuous variables. To test for between-group differences in baseline and follow-up daily sunscreen use in the past 30 days, we will use a cluster-adjusted Mantel-Haenszel χ^2 test [66] with a significance level of 5% (two-sided). For the main analysis, HLM (hierarchical linear models) will be applied. HLM can handle the nested structure of the data and will be used to test for between-group differences in within-group changes in sun protection behavior over time. HLM will also be used to investigate the influence of further

covariates (such as gender, European ancestry and skin type) and time-dependent behavior in secondary analyses. Statistical analyses will be performed using SPSS Statistics (IBM Corporation, New York, USA).

The effect that missing data may have on results will be assessed via sensitivity analysis. Dropouts (essentially participants who withdraw consent for continued follow-up or who are missing from the classroom during the survey) will be included in the analysis, and multiple imputation will be used to estimate the treatment effect [67].

Discussion

This is the first cluster-randomized school-based trial on photoaging skin cancer prevention and the first trial on medical student-delivered school-based skin cancer prevention worldwide. While classic health educational school-based approaches in skin cancer prevention were evaluated as inferior to appearance-based approaches [19], there is a global lack of novel, innovative strategies that harness current technology while taking widely accepted theories for behavioral change into account [61]. Although multiple studies have shown that skin cancer risk is predominantly associated with sun exposure early in life, there is often a lack of awareness in risk groups.

Thus, this trial provides the opportunity to evaluate an innovative, highly scalable, appearance-based intervention in a high-risk population. It will also provide data to estimate whether photoaging mobile apps have the potential to be broadly implemented in schools via the mirroring intervention but also via other avenues (i.e., posters [65] or smartphone-based advertising campaigns in the App Store and Google Play Store) or could be a valuable addition to existing educational programs.

Additionally, because it is delivered by medical students, this trial also sensitizes future physicians to the importance of skin cancer prevention, highlighting their associated responsibilities within communities [35, 68].

According to the theory of planned behavior (TPB), the subjective norm and the expected self-efficacy of the participants play a substantial role in their resulting behavior. For example: What do their peers think about tanning? Is the result of tanning regarded as attractive and does it therefore increase one's chances of finding a boy/girlfriend? How likely is it that a behavioral change can positively influence this reaction? The mirroring intervention triggers strong reactions of the peer group of the individual participant towards their photoaged future self (=affecting subjective norm) but also illustrates the power of one's own behavioral change (and thereby increases one's expectation of self-efficacy, another predictor of the TPB) to influence this reaction by peers [49].

Limitations

Because this study is conducted only in Brazil, the results may not be generalizable to other cultural or national settings. However, the theoretical basis for this intervention (the theory of planned behavior) has been proven to apply to most cultural contexts around the globe [61]. As this trial enrolls approximately 10 different public schools, it should be representative for most school types.

We must choose classes and not schools as a cluster due to sample size limitations; thus, cluster effects cannot be entirely excluded. However, multiple steps are taken to limit contamination between the control and intervention classes (i.e., the name of the app is not mentioned to the pupils by the trained medical students, and the teachers of the control classes are strictly prohibited to talk about the intervention

with their students). Cluster effects are also monitored in the endline questionnaire and provide a basis for a sensitivity analysis.

Some students may find the effects of the Sunface App unrealistic, as indicated in our recently published pilot study. However, this does not appear to attenuate motivation to adhere to UV protection behavior [60].

In summary, we evaluate the long-term effects on behavior of a novel method that integrates photoaging in a school-based skin cancer prevention program in a population with a high risk for developing skin cancer. The program affects the students' peer group and also considers predictors for tanning.

Ethics and Dissemination

Participation is voluntary and oral consent is sufficient. We are asking for this Ethics Committee to waive the necessity for informed written consent because: 1) this is not a biomedical intervention itself (ie, the main focus of the project is educational, and the project does not include any medication, laboratory test, or biological material); 2) the information collected will be non-identifable (identification of the same respondents at each time point will be made with a self-generated identification code, permitting an anonymous means to track respondents over the multiple data collection points). All participant information will be stored in locked file cabinets in areas with limited access. Results will be disseminated at conferences, in peer-reviewed journals and on our websites.

Conflicts of Interest

None declared.

Funding

The tablets are funded by the Young Research Award Research Grant from La Fondation La Roche Posay awarded to Titus J. Brinker for his research on the Sunface app. The University of Itauna will contribute by providing logistic support for the project and copies of all questionnaires. La Fondation La Roche Posay and the University of Itauna Funding Board had no role in the design and conduction of this study or in the preparation or review of this project.

References

1. Livingstone E, Windemuth-Kieselbach C, Eigentler TK, Rompel R, Trefzer U, Nashan D, Rotterdam S, Ugurel S, Schadendorf D: **A first prospective population-based analysis investigating the actual practice of melanoma diagnosis, treatment and follow-up**. *European Journal of Cancer* 2011, **47**(13):1977-1989.
2. The Lancet Editorial Board: **Skin cancer: prevention is better than cure**. *The Lancet* 2014, **384**(9942):470.
3. Green AC, Williams GM, Logan V, Strutton GM: **Reduced melanoma after regular sunscreen use: randomized trial follow-up**. *Journal of Clinical Oncology* 2010, **29**(3):257-263.
4. Ghiasvand R, Weiderpass E, Green AC, Lund E, Veierød MB: **Sunscreen use and subsequent melanoma risk: A population-based cohort study**. *Journal of Clinical Oncology* 2016, **34**(33):3976-3983.
5. Nijsten T: **Sunscreen Use in the Prevention of Melanoma: Common Sense Rules**. In.: American Society of Clinical Oncology; 2016.
6. Ou-Yang H, Jiang LI, Meyer K, Wang SQ, Farberg AS, Rigel DS: **Sun Protection by Beach Umbrella vs Sunscreen With a High Sun Protection Factor: A Randomized Clinical Trial**. *JAMA dermatology* 2017, **153**(3):304-308.
7. Benvenuto ‐ Andrade C, Zen B, Fonseca G, Villa D, Cestari T: **Sun Exposure and Sun Protection Habits Among High ‐ school Adolescents in Porto Alegre, Brazil**. *Photochemistry and photobiology* 2005, **81**(3):630-635.
8. Purim KSM, Wroblevski FC: **Sun exposure and protection among medical students in curitiba (PR)**. *Revista Brasileira de Educação Médica* 2014, **38**(4):477-485.
9. Schalka S, Steiner D, Ravelli FN, Steiner T, Terena AC, Marçon CR, Ayres EL, Addor FASa, Miot HA, Ponzio H: **Brazilian consensus on photoprotection**. *Anais brasileiros de dermatologia* 2014, **89**(6):1-74.
10. Haack RL, Horta BL, Cesar JA: **Sunburn in young people: population-based study in Southern Brazil**. *Revista de Saúde Pública* 2008, **42**(1):26-33.

11. Silva AA: **Outdoor Exposure to Solar Ultraviolet Radiation and Legislation in Brazil**. *Health physics* 2016, **110**(6):623-626.
12. Lima ASd, Stein CE, Casemiro KP, Rovere RK: **Epidemiology of Melanoma in the South of Brazil: study of a city in the Vale do Itajaí from 1999 to 2013**. *Anais brasileiros de dermatologia* 2015, **90**(2):185-189.
13. de Lima Vazquez V, Silva TB, de Andrade Vieira M, de Oliveira ATT, Lisboa MV, de Andrade DAP, Fregnani JHTG, Carneseca EC: **Melanoma characteristics in Brazil: demographics, treatment, and survival analysis**. *BMC research notes* 2015, **8**(1):4.
14. Amancio CT, Nascimento LFC: **Cutaneous melanoma in the State of São Paulo: a spatial approach**. *Anais brasileiros de dermatologia* 2014, **89**(3):442-446.
15. Naser N: **Cutaneous melanoma: a 30-year-long epidemiological study conducted in a city in southern Brazil, from 1980-2009**. *Anais brasileiros de dermatologia* 2011, **86**(5):932-941.
16. Lo JA, Fisher DE: **The melanoma revolution: from UV carcinogenesis to a new era in therapeutics**. *Science* 2014, **346**(6212):945-949.
17. Wu S, Han J, Laden F, Qureshi AA: **Long-term ultraviolet flux, other potential risk factors, and skin cancer risk: a cohort study**. *Cancer Epidemiology and Prevention Biomarkers* 2014, **23**(6):1080-1089.
18. Barton MK: **Indoor tanning increases melanoma risk, even in the absence of a sunburn**. *CA: a cancer journal for clinicians* 2014, **64**(6):367-368.
19. Tuong W, Armstrong AW: **Effect of appearance-based education compared with health-based education on sunscreen use and knowledge: a randomized controlled trial**. *Journal of the American Academy of Dermatology* 2014, **70**(4):665-669.
20. Craciun C, Schüz N, Lippke S, Schwarzer R: **Facilitating sunscreen use in women by a theory-based online intervention: A randomized controlled trial**. *Journal of health psychology* 2012, **17**(2):207-216.
21. Hirst NG, Gordon LG, Scuffham PA, Green AC: **Lifetime cost-effectiveness of skin cancer prevention through promotion of daily sunscreen use**. *Value in Health* 2012, **15**(2):261-268.
22. Sakoufaki M, Stergiopoulou A, Stratigos A: **Design and implementation of a health promotion program to prevent the harmful effects of ultraviolet radiation at primary school students of rural areas of Greece**. *International Journal of Research in Dermatology* 2017.
23. Miller K, Langholz B, Ly T, Harris S, Richardson J, Peng D, Cockburn M: **SunSmart: evaluation of a pilot school-based sun protection intervention in Hispanic early adolescents**. *Health education research* 2015:cyv011.
24. Aarestrup C, Bonnesen CT, Thygesen LC, Krarup AF, Waagstein AB, Jensen PD, Bentzen J: **The effect of a school-based intervention on sunbed use in Danish pupils at continuation schools: a cluster-randomized controlled trial**. *Journal of Adolescent Health* 2014, **54**(2):214-220.
25. Holman DM, Fox KA, Glenn JD, Guy GP, Watson M, Baker K, Cokkinides V, Gottlieb M, Lazovich D, Perna FM: **Strategies to reduce indoor tanning: current research gaps and future opportunities for prevention**. *American journal of preventive medicine* 2013, **44**(6):672-681.
26. Hillhouse J, Turrisi R, Scaglione NM, Cleveland MJ, Baker K, Florence LC: **A Web-Based Intervention to Reduce Indoor Tanning Motivations in Adolescents: a Randomized Controlled Trial**. *Prevention Science* 2016:1-10.

27. Stapleton JL, Hillhouse J, Levonyan-Radloff K, Manne SL: **Review of Interventions to Reduce Ultraviolet Tanning: Need for Treatments Targeting Excessive Tanning, An Emerging Addictive Behavior**. 2017.
28. Olson AL, Gaffney C, Starr P, Gibson JJ, Cole BF, Dietrich AJ: **SunSafe in the middle school years: a community-wide intervention to change early-adolescent sun protection**. *Pediatrics* 2007, **119**(1):e247-e256.
29. Miller KA, Huh J, Unger JB, Richardson JL, Allen MW, Peng DH, Cockburn MG: **Patterns of sun protective behaviors among Hispanic children in a skin cancer prevention intervention**. *Preventive medicine* 2015, **81**:303-308.
30. Turner D, Harrison SL, Buettner P, Nowak M: **Does being a "SunSmart School" influence hat-wearing compliance? An ecological study of hat-wearing rates at Australian primary schools in a region of high sun exposure**. *Preventive medicine* 2014, **60**:107-114.
31. Buller DB, Andersen PA, Walkosz BJ, Scott MD, Beck L, Cutter GR: **Rationale, design, samples, and baseline sun protection in a randomized trial on a skin cancer prevention intervention in resort environments**. *Contemporary clinical trials* 2016, **46**:67-76.
32. Sontag JM, Noar SM: **Assessing the Potential Effectiveness of Pictorial Messages to Deter Young Women from Indoor Tanning: An Experimental Study**. *Journal of Health Communication* 2017, **22**(4):294-303.
33. Guy GP, Holman DM, Watson M: **The Important Role of Schools in the Prevention of Skin Cancer**. *Jama dermatology* 2016, **152**(10):1083-1084.
34. Farberg AS, Glazer AM, Rigel AC, White R, Rigel DS: **Dermatologists' Perceptions, Recommendations, and Use of Sunscreen**. *Jama dermatology* 2017, **153**(1):99-101.
35. Isvy A, Beauchet A, Saiag P, Mahé E: **Medical students and sun prevention: knowledge and behaviours in France**. *Journal of the European Academy of Dermatology and Venereology* 2013, **27**(2):e247-e251.
36. Nanyes JE, McGrath JM, Krejci-Manwaring J: **Medical Students' Perceptions of Skin Cancer: Confusion and Disregard for Warnings and the Need for New Preventive Strategies**. *Archives of dermatology* 2012, **148**(3):392-393.
37. Patel SS, Nijhawan RI, Stechschulte S, Parmet Y, Rouhani P, Kirsner RS, Hu S: **Skin cancer awareness, attitude, and sun protection behavior among medical students at the University of Miami Miller School of Medicine**. *Archives of dermatology* 2010, **146**(7):797-800.
38. Brinker TJ, Stamm-Balderjahn S, Seeger W, Klingelhöfer D, Groneberg DA: **Education Against Tobacco (EAT): a quasi-experimental prospective evaluation of a multinational medical-student-delivered smoking prevention programme for secondary schools in Germany**. *BMJ open* 2015, **5**(9):e008093.
39. Brinker TJ, Stamm-Balderjahn S, Seeger W, Groneberg DA: **Education Against Tobacco (EAT): a quasi-experimental prospective evaluation of a programme for preventing smoking in secondary schools delivered by medical students: a study protocol**. *BMJ Open* 2014, **4**(7):e004909.
40. Görig T, Diehl K, Greinert R, Breitbart EW, Schneider S: **Prevalence of sun-protective behaviour and intentional sun tanning in German adolescents and adults: results of a nationwide telephone survey**. *Journal of the European Academy of Dermatology and Venereology* 2017, **Volume 31**(Issue 6):Pages 913–1077, e1273–e1306.

41. Schneider S, Diehl K, Bock C, Schlüter M, Breitbart EW, Volkmer B, Greinert R: **Sunbed use, user characteristics, and motivations for tanning: results from the German population-based SUN-Study 2012**. *JAMA dermatology* 2013, **149**(1):43-49.
42. Hillhouse J, Turrisi R, Scaglione NM, Cleveland MJ, Baker K, Florence LC: **A Web-Based Intervention to Reduce Indoor Tanning Motivations in Adolescents: a Randomized Controlled Trial**. *Prevention Science* 2017, **18**(2):131-140.
43. Hillhouse J, Turrisi R, Cleveland MJ, Scaglione NM, Baker K, Florence LC: **Theory-driven longitudinal study exploring indoor tanning initiation in teens using a person-centered approach**. *Annals of Behavioral Medicine* 2016, **50**(1):48-57.
44. Baudson TG, Weber KE, Freund PA: **More Than Only Skin Deep: Appearance Self-Concept Predicts Most of Secondary School Students' Self-Esteem**. *Frontiers in psychology* 2016, **7**.
45. Wichstrøm L, von Soest T: **Reciprocal relations between body satisfaction and self-esteem: A large 13-year prospective study of adolescents**. *Journal of adolescence* 2016, **47**:16-27.
46. Bränström R, Kasparian NA, Chang Y-m, Affleck P, Tibben A, Aspinwall LG, Azizi E, Baron-Epel O, Battistuzzi L, Bergman W: **Predictors of sun protection behaviors and severe sunburn in an international online study**. *Cancer Epidemiology and Prevention Biomarkers* 2010, **19**(9):2199-2210.
47. Görig T, Diehl K, Greinert R, Breitbart E, Schneider S: **Prevalence of sun‐protective behavior and intentional sun tanning in German adolescents and adults: results of a nationwide telephone survey**. *Journal of the European Academy of Dermatology and Venereology* 2017.
48. Brinker TJ, Seeger W: **Photoaging Mobile Apps: A Novel Opportunity for Smoking Cessation?** *Journal of medical Internet research* 2015, **17**(7):e186.
49. Brinker TJ, Seeger W, Buslaff F: **Photoaging Mobile Apps in School-Based Tobacco Prevention: The Mirroring Approach**. *J Med Internet Res* 2016, **18**(6):e183.
50. Burford O, Jiwa M, Carter O, Parsons R, Hendrie D: **Internet-Based Photoaging Within Australian Pharmacies to Promote Smoking Cessation: Randomized Controlled Trial**. *J Med Internet Res* 2013, **15**(3):e64.
51. Jiwa M, Burford O, Parsons R: **Preliminary findings of how visual demonstrations of changes to physical appearance may enhance weight loss attempts.** *The European Journal of Public Health* 2015, **25**(2):283-285.
52. Brinker TJ, Owczarek AD, Seeger W, Groneberg DA, Brieske CM, Jansen P, Klode J, Stoffels I, Schadendorf D, Izar B: **A Medical Student-Delivered Smoking Prevention Program, Education Against Tobacco, for Secondary Schools in Germany: Randomized Controlled Trial.** *Journal of Medical Internet Research* 2017, **19**(6):e199.
53. Brinker TJ, Brieske CM, et al.: **A Dermatologists' Ammunition in the War Against Smoking.** *J Med Internet Res* 2017, **forthcoming**.
54. Williams AL, Grogan S, Clark-Carter D, Buckley E: **Impact of a facial-ageing intervention versus a health literature intervention on women's sun protection attitudes and behavioural intentions.** *Psychology & health* 2013, **28**(9):993-1008.
55. Presti LL, Chang P, Taylor MF: **Young Australian adults' reactions to viewing personalised UV photoaged photographs**. *The Australasian medical journal* 2014, **7**(11):454.

56. Robinson JK, Wayne JD, Martini MC, Hultgren BA, Mallett KA, Turrisi R: **Early detection of new melanomas by patients with melanoma and their partners using a structured skin self-examination skills training intervention: a randomized clinical trial.** *JAMA dermatology* 2016, **152**(9):979-985.
57. Brinker TJ, Schadendorf D, Klode J, Cosgarea I, Rösch A, Jansen P, Stoffels I, Izar B: **Photoaging Mobile Apps as a Novel Opportunity for Melanoma Prevention: Pilot Study**. *JMIR mHealth and uHealth* 2017, **5**(7):e101.
58. D'Orazio J, Jarrett S, Amaro-Ortiz A, Scott T: **UV radiation and the skin**. *International journal of molecular sciences* 2013, **14**(6):12222-12248.
59. Kammeyer A, Luiten R: **Oxidation events and skin aging**. *Ageing research reviews* 2015, **21**:16-29.
60. Brinker TJ, Brieske CM, Schaefer CM, Buslaff F, Gatzka M, Petri MP, Sondermann W, Schadendorf D, Stoffels I, Klode J: **Photoaging Mobile Apps in School-Based Melanoma Prevention: Pilot Study**. *Journal of Medical Internet Research* 2017, **19**(9):e319.
61. Ajzen I: **Theory of planned behavior**. *Handb Theor Soc Psychol Vol One* 2011, **1**:438.
62. Bakos L, Masiero N, Bakos R, Burttet R, Wagner M, Benzano D: **European ancestry and cutaneous melanoma in Southern Brazil.** *Journal of the European Academy of Dermatology and Venereology* 2009, **23**(3):304-307.
63. Detert H, Hedlund S, Anderson C, Rodvall Y, Festin K, Whiteman D, Falk M: **Validation of sun exposure and protection index (SEPI) for estimation of sun habits**. *Cancer epidemiology* 2015, **39**(6):986-993.
64. Robine J, Jagger C: **Translation & Linguistic Evaluation protocol & Supporting Material**. *WHO/UNESCAP Project on Health and Disability Statistics* 2003.
65. Brinker TJ, Holzapfel J, Baudson TG, Sies K, Jakob L, Baumert HM, Heckl M, Cirac A, Suhre JL, Mathes V: **Photoaging smartphone app promoting poster campaign to reduce smoking prevalence in secondary schools: the Smokerface Randomized Trial: design and baseline characteristics**. *BMJ open* 2016, **6**(11):e014288.
66. Donald A, Donner A: **Adjustments to the Mantel‐Haenszel chi‐square statistic and odds ratio variance estimator when the data are clustered**. *Statistics in Medicine* 1987, **6**(4):491-499.
67. Sterne JA, White IR, Carlin JB, Spratt M, Royston P, Kenward MG, Wood AM, Carpenter JR: **Multiple imputation for missing data in epidemiological and clinical research: potential and pitfalls**. *Bmj* 2009, **338**:b2393.
68. Rat C, Houd S, Gaultier A, Grimault C, Quereux G, Mercier A, Letrilliart L, Dreno B, Nguyen JM: **General practitioner management related to skin cancer prevention and screening during standard medical encounters: a French cross-sectional study based on the International Classification of Primary Care**. *BMJ open* 2017, **7**(1):e013033.

Appendix – Questionnaire

Dear student,

Surveys about sun exposure are conducted in several schools across Brazil. Your participation in this survey is voluntary and this questionnaire is **anonymous**. Accordingly, please give **honest answers**, otherwise the whole survey makes no sense. Nobody else than the researchers conducting this survey will see your questionnaire. And even the researchers do not know who you are as they can see only your code. **Consequently, nobody will know that you filled out this questionnaire.** Please start by creating your **personal code**:

① Take the **first** and the **last** letter of the first name of your mother (in case your mother`s name was **A**nn**e** this would be **AE)**

② Afterwards, take your **day** of birth (if it was 5th of March it would be **05**)

③ Finally, take the the **first** and the **last** letter of **your first name** (if your name is **R**afae**l**, this would be **RL**)

In this example your Code would be **A E 0 5 R L**

Please now enter **your personal code** in the same manner into these boxes:

☐ ☐ ☐ ☐ ☐ ☐

Today's date: ____/____/_____

There is no "right" or "wrong" answer for the questions. Please answer each question based on your own judgment and not based on how you think others would like you to answer it. Read the questionnaire carefully before answering, but trust your first thought when ticking a box - the first response that crosses our mind is usually the most appropriate one!

Check only one box for each question!

1) Gender: ☐ male ☐ female

2) Your age (in years): ☐10 ☐11 ☐12 ☐13 ☐14 ☐15 ☐16 ☐17 ☐18 ☐19 ☐20

3) Was at least one of your parents or grandparents born in Europe? ☐yes ☐no

4) I use a smartphone: ☐yes ☐no

5) During the past 30 days, on how many days did you use sunscreen?

☐ Daily
☐ Almost daily
☐ Sometimes
☐ Rarely
☐ Never

6) Do you think a tan looks attractive?

☐ yes
☐ no
☐ not sure

7) Do you think tanning makes you look attractive?

☐ yes
☐ no
☐ not sure

8) Overall, would you say tanning is healthy for you?

☐ yes
☐ no
☐ not sure

9) What is the most important reason for you to tan?

☐ It is healthy for me
☐ It makes me look more attractive
☐ It helps me to relax
☐ Other reason that is not listed here
☐ I do not tan

10) How often do you tan?

☐ At least once a month
☐ At least once every 3 months
☐ At least once every 6 months
☐ At least once a year
☐ I do not tan

11) How do you usually tan?

☐ In a sunbed
☐ In the sun, using a tanning lotion that contains sunscreen
☐ In the sun, using a tanning lotion that does not contain sunscreen
☐ In the sun, without tanning lotion
☐ I do not tan

☐ I tan using other method. **11.2)** Which? ______________________________

12) Have you used the sunbed at least once in your life? ☐ yes ☐ no

13) Have you had a sunburn at least once in your life? ☐ yes ☐ no

14) Have you had a sunburn with blistering at least once in your life? ☐ yes ☐ no

15) Your eye color is:

☐ Light blue, light gray or light green
☐ Blue, gray or green
☐ Hazel or light brown
☐ Dark brown
☐ Brownish black

16) Your natural hair color is:

☐ Red or light blonde
☐ Blonde
☐ Dark blonde or light brown
☐ Dark brown
☐ Black

17) Your natural skin color (before sun exposure) is:

☐ Ivory white
☐ Fair or pale
☐ Fair to beige, with golden undertone
☐ Olive or light brown
☐ Dark brown or black

18) How many freckles do you have on unexposed areas of your skin?

☐ Many
☐ Several
☐ A few
☐ Very few
☐ None

19) How does your skin respond to the sun?

☐ Always burns, blisters and peels
☐ Often burns, blisters and peels
☐ Burns moderately
☐ Burns rarely, if at all
☐ Never burns

20) Does your skin tan?

☐ Never -- I always burn
☐ Seldom
☐ Sometimes
☐ Often
☐ Always

21) How deeply do you tan?

☐ Not at all or very little
☐ Lightly
☐ Moderately
☐ Deeply
☐ My skin is naturally dark

22) How sensitive is your face to the sun?

☐ Very sensitive
☐ Sensitive
☐ Normal
☐ Resistant
☐ Very resistant/Never had a problem

23) How often do you sunbathe with the intention to get tanned?

☐ Never
☐ Seldom
☐ Occasionally
☐ Often
☐ Always

24) How many times have you been sunburnt (redness and smarting pain) during the last 12 months?

☐ None
☐ 1-2 times
☐ 3-5 times
☐ 6-10 times
☐ More than 10 times

25) How long do you usually stay in the sun (on average), between 11 am and 3 pm, on a typical day-off?

☐ < 30 min
☐ 30 min – 1 hour
☐ 1-2 hours
☐ 2-3 hours
☐ > 3 hours

26) How often do you take a holiday with the intention of spending more time in the sun?

☐ Never
☐ Seldom
☐ 1-2 weeks a year
☐ 3-5 weeks a year
☐ > 5 weeks a year

27) When in the sun, how often do you use sunscreens?

☐ Always
☐ Often
☐ Occasionally
☐ Seldom
☐ Never

28) When in the sun, how often do you use covering clothes for sun protection?

☐ Always
☐ Often
☐ Occasionally
☐ Seldom
☐ Never

29) When in the sun, how often do you use a sun hat or cap for sun protection?

☐ Always
☐ Often
☐ Occasionally
☐ Seldom
☐ Never

30) How often do you stay indoors or in the shade in order to protect yourself from the sun?

☐ Always
☐ Often
☐ Occasionally
☐ Seldom
☐ Never

31) During the past 6 months, how many times did you examine your skin looking for alterations (such as unusual spots, skin cancer)?

☐ not at all
☐ once
☐ more than once

32) How many tanning sessions did you have in the past 30 days?

☐ None
☐ 1 session
☐ 2 sessions
☐ 3 sessions
☐ 4 sessions
☐ 5 or more sessions
