# Supplementary material for: Effect of a Face-Aging Mobile App–Based Intervention on Skin Cancer Protection Behavior in Secondary Schools in Brazil: A Cluster-Randomized Clinical Trial
Source: JAMA Dermatol. 2020 May 6;156(7):737–45. doi: 10.1001/jamadermatol.2020.0511 (PMC7203674; doi:10.1001/jamadermatol.2020.0511)
Supplement: Supplement 2. — eTable 1. Intraclass Correlation Coefficients (ICCs) Determined by the ANOVA Method eTable 2. Bivariate Relations at Baseline (All Cases Including Dropouts) eTable 3. Perception of Students on the Intervention–Immediate Postintervention Survey eTable 4. Descriptive Characteristics of Pupils With Daily Use of Sunscreen During the Past 30 Days at Baseline and 3- and 6-Month Follow-up eTable 5. Comparison of the Change of Daily Sunscreen Use Between the Intervention and Control Groups eTable 6. Gender-Specific Comparison of the Change of Daily Sunscreen Use Between the Intervention and Control Groups eTable 7. Analysis of the Influence of Different Factors on the Primary End Point eTable 8. Analysis of the Influence of Different Factors on the Secondary End Point “Daily Sunscreen Use at 3-Month Follow-up” eTable 9. Comparison of the Change of Daily Sunscreen Use Between the Intervention and Control Groups eTable 10. Gender-Specific Comparison of the Change of Daily Sunscreen Use Between the Intervention and Control Groups eTable 11. Descriptive Characteristic of Pupils With at Least 1 Skin Self-examination in the Past 6 Months at Baseline and 3- and 6-Month Follow-up eTable 12. Comparison of the Change of the Prevalence of Pupils With at Least 1 Skin Self-examination Between the Intervention and Control Groups eTable 13. Gender-Specific Comparison of the Change of the Prevalence of Pupils With at Least 1 Skin Self-examination Between the Intervention and Control Groups eTable 14. Analysis of the Influence of Different Factors on the Secondary End Point “Skin Self-examination Within the Past 6 Months” eTable 15. Descriptive Characteristic of Pupils With at Least 1 Tanning Session in the Past 30 Days at Baseline and 3- and 6-Month Follow-up eTable 16. Comparison of the Change of the Prevalence of Pupils With at Least 1 Tanning Session in the Past 30 Days Between the Intervention and Control Groups eTable 17. Gender-Specific Comparison of the Change of the Prevalence of P [file jamadermatol-156-737-s002.pdf]

# Supplementary Online Content

Brinker TJ, Faria BL, de Faria OM, et al. Effect of a face-aging mobile app–based intervention on skin cancer protection behavior in secondary schools in Brazil: a cluster-randomized clinical trial. *JAMA Dermatol*. Published online May 6, 2020. doi:10.1001/jamadermatol.2020.0511

**eTable 1.** Intraclass Correlation Coefficients (ICCs) Determined by the ANOVA Method

**eTable 2.** Bivariate Relations at Baseline (All Cases Including Dropouts)

**eTable 3.** Perception of Students on the Intervention–Immediate Postintervention Survey

**eTable 4.** Descriptive Characteristics of Pupils With Daily Use of Sunscreen During the Past 30 Days at Baseline and 3- and 6-Month Follow-up

**eTable 5.** Comparison of the Change of Daily Sunscreen Use Between the Intervention and Control Groups

**eTable 6.** Gender-Specific Comparison of the Change of Daily Sunscreen Use Between the Intervention and Control Groups

**eTable 7.** Analysis of the Influence of Different Factors on the Primary End Point

**eTable 8.** Analysis of the Influence of Different Factors on the Secondary End Point “Daily Sunscreen Use at 3-Month Follow-up”

**eTable 9.** Comparison of the Change of Daily Sunscreen Use Between the Intervention and Control Groups

**eTable 10.** Gender-Specific Comparison of the Change of Daily Sunscreen Use Between the Intervention and Control Groups

**eTable 11.** Descriptive Characteristic of Pupils With at Least 1 Skin Self-examination in the Past 6 Months at Baseline and 3- and 6-Month Follow-up

**eTable 12.** Comparison of the Change of the Prevalence of Pupils With at Least 1 Skin Self-examination Between the Intervention and Control Groups

**eTable 13.** Gender-Specific Comparison of the Change of the Prevalence of Pupils With at Least 1 Skin Self-examination Between the Intervention and Control Groups

**eTable 14.** Analysis of the Influence of Different Factors on the Secondary End Point “Skin Self-examination Within the Past 6 Months”

**eTable 15.** Descriptive Characteristic of Pupils With at Least 1 Tanning Session in the Past 30 Days at Baseline and 3- and 6-Month Follow-up

**eTable 16.** Comparison of the Change of the Prevalence of Pupils With at Least 1 Tanning Session in the Past 30 Days Between the Intervention and Control Groups

**eTable 17.** Gender-Specific Comparison of the Change of the Prevalence of Pupils With at Least 1 Tanning Session in the Past 30 Days Between the Intervention and Control Groups

**eTable 18.** Analysis of the Influence of Different Factors on the Secondary End Point “at Least 1 Tanning Session Within the Past 30 Days”

**eTable 19.** Dropouts in Relation to Assigned Group

**eTable 20.** Dropouts in Relation to Sunscreen Use

**eTable 21.** Dropouts in Relation to Skin Self-examinations

**eTable 22.** Dropouts in Relation to Tanning Sessions

**eTable 23.** Dropouts in Relation to Gender

This supplementary material has been provided by the authors to give readers additional information about their work.

©2020 Brinker TJ et al. *JAMA Dermatology*.

## Baseline analyses

### eTable 1. Intraclass Correlation Coefficents (ICCs) Determined by the ANOVA Method

| Cluster Level | Endpoint | ICC value |
|---|---|---|
| School | Daily sunscreen use in past 30 days | 0.0016 |
| School | Self-skin examination in last 6 months | 0.0120 |
| School | Tanning sessions in past 30 days | 0.0339 |
| Class | Daily sunscreen use in past 30 days | 0.0066 |
| Class | Self-skin examination in last 6 months | 0.0185 |
| Class | Tanning sessions in past 30 days | 0.0538 |

(Zou & Donner, 2004).

**eTable 2. Bivariate Relations at Baseline (All Cases Including Dropouts)**

| | | Daily sunscreen use in past 30 days | Self-skin examination in last 6 months | Tanning sessions in past 30 days |
|---|---|---|---|---|
| | | Category: Yes | Once or more | One at least |
| **Total sample (N=1573)** | | 14.9% | 27.0% | 15.7% |
| **Gender** | Female (N=812) | 20.8% | 30.3% | 21.1% |
| | Male (N=761) | 8.7% | 23.5% | 10.0% |
| | Odds Ratio | 0.36 | 0.71 | 0.42 |
| | Significance | p=0.000 | p=0.003 | p=0.000 |
| **School grade** | 9th grade (N=258) | 14.3% | 30.2% | 20.2% |
| | 10th grade (N=452) | 13.1% | 24.6% | 14.8% |
| | 11th grade (N=424) | 15.3% | 26.9% | 15.8% |
| | 12th grade (N=439) | 16.9% | 27.8% | 13.9% |
| | Odds Ratio | 1.10 | 0.99 | 0.89 |
| | Significance | p=0.179 | p=0.911 | p=0.077 |

Odds ratios and significances were computed using Binary Logistic Regression with targets as dependent dichotomous variables.

©2020 Brinker TJ et al. *JAMA Dermatology*.

**eTable 3. Perception of Students on the Intervention–Immediate Postintervention Survey**

Immediately after the intervention was finished, the perception of the students was measured anonymously via a questionnaire with 4 items on a 5-point Likert scale.

| **Item** | **Strongly agree or agree n/N(%)** | **Neutral n/N(%)** | **Strongly disagree or disagree n/N(%)** |
|---|---|---|---|
| "The intervention was fun" | 690/734 (94.0%) | 23/734 (3.1%) | 21/734 (2.9%) |
| "I learned new benefits of sun protection" | 701/734 (95.5%) | 30/734 (4.1%) | 3/734 (0.4%) |
| "The animation of my 3D selfie motivates me to use daily sunscreen" | 662/734 (90.2%) | 57/734 (7.8%) | 15/734 (2.0%) |
| "The intervention motivates me to check my skin with the ABCDE rule in the next 6 months" | 668/734 (91.0%) | 51/734 (7.0%) | 15/734 (2.0%) |

## Primary end point: daily sunscreen use at 6 months follow-up

### eTable 4. Descriptive Characteristics of Pupils With Daily Use of Sunscreen During the Past 30 Days at Baseline and 3- and 6-Month Follow-up

95% confidence intervals were calculated using the Clopper and Pearson method.

| | | Time Point* | Intervention Group | 95%CI | Control Group | 95%CI |
|---|---|---|---|---|---|---|
| Total | | B | 110/734 (15.0%) | 12.5 – 17.8 % | 125/839 (14.9%) | 12.6 – 17.5 % |
| | | F3 | 136/618 (22.0%) | 18.8 – 25.5 % | 103/784 (13.1%) | 10.9 – 15.7 % |
| | | F6 | 139/607 (22.9%) | 19.6 – 26.5 % | 114/787 (14.5%) | 12.1 – 17.1 % |
| Gender | Male | B | 31/336 (9.2%) | 6.4 – 12.8 % | 35/425 (8.2%) | 5.8 – 11.3 % |
| | | F3 | 28/275 (10.2%) | 6.9 – 14.4 % | 22/395 (5.6%) | 3.5 – 8.3 % |
| | | F6 | 31/269 (11.5%) | 8.0 – 16.0 % | 30/392 (7.7%) | 5.2 – 10.7 % |
| | Female | B | 79/398 (19.8%) | 16.0 – 24.1 % | 90/414 (21.7%) | 17.9 – 26.0 % |
| | | F3 | 108/343 (31.5%) | 26.6 – 36.7 % | 81/389 (20.8%) | 16.9 – 25.2 % |
| | | F6 | 108/338 (32.0%) | 27.0 – 37.2 % | 84/395 (21.3%) | 17.3 – 25.6 % |
| School grade | 9th grade | B | 14/145 (9.7%) | 5.4 – 15.7 % | 23/113 (20.4%) | 13.4 – 29.0 % |
| | | F3 | 27/126 (21.4%) | 14.6 – 29.6 % | 17/108 (15.7%) | 9.4 – 24.0 % |
| | | F6 | 28/126 (22.2%) | 15.3 – 30.5 % | 20/108 (18.5%) | 11.7 – 27.1 % |
| | 10th grade | B | 33/221 (14.9%) | 10.5 – 20.3 % | 26/231 (11.3%) | 7.5 – 16.1 % |
| | | F3 | 43/181 (23.8%) | 17.8 – 30.6 % | 22/218 (10.1%) | 6.4 – 14.9 % |
| | | F6 | 41/189 (21.7%) | 16.0 – 28.3 % | 25/213 (11.7%) | 7.7 – 16.8 % |
| | 11th grade | B | 24/178 (13.5%) | 8.8 – 19.4 % | 41/246 (16.7%) | 12.2 – 21.9 % |
| | | F3 | 23/149 (15.4%) | 10.0 – 22.3 % | 33/231 (14.3%) | 10.0 – 19.5 % |
| | | F6 | 28/147 (19.0%) | 13.0 – 26.3 % | 39/237 (16.5%) | 12.0 – 21.8 % |
| | 12th grade | B | 39/190 (20.5%) | 15.0 – 27.0 % | 35/249 (14.1%) | 10.0 – 19,0 % |
| | | F3 | 43/162 (26.5%) | 19.9 – 34.0 % | 31/227 (13.7%) | 9.5 – 18.8 % |
| | | F6 | 42/145 (29.0%) | 21.7 – 37.1 % | 30/229 (13.1%) | 9.0 – 18.2 % |
| Fitzpatrick Skin Type | Type I or II | B | 11/46 (23.9%) | 12.6 – 38.8 % | 18/70 (25.7%) | 16.0 – 37.6 % |
| | | F3 | 12/39 (30.8%) | 17.0 – 47.6 % | 14/64 (21.9%) | 12.5 – 34.0 % |
| | | F6 | 14/37 (37.8%) | 22.5 – 55.2 % | 14/65 (21.5%) | 12.3 – 33.5 % |
| | Type III | B | 43/256 (16.8%) | 12.4 – 22.0 % | 42/293 (14.3%) | 10.5 – 18.9 % |
| | | F3 | 51/210 (24.3%) | 18.6 – 30.7 % | 45/275 (16.4%) | 12.2 – 21.3 % |
| | | F6 | 56/211 (26.5%) | 20.7 – 33.0 % | 46/273 (16.8%) | 12.6 – 21.8 % |
| | Type IV | B | 49/368 (13.3%) | 10.0 – 17.2 % | 56/421 (13.3%) | 10.2 – 16.9 % |
| | | F3 | 67/316 (21.2%) | 16.8 – 26.1 % | 36/393 (9.2%) | 6.5 – 12.5 % |
| | | F6 | 61/311 (19.6%) | 15.3 – 24.5 % | 45/396 (11.4%) | 8.4 – 14.9 % |
| | Type V | B | 7/64 (10.9%) | 4.5 – 21.2 % | 9/55 (16.4%) | 7.8 – 28.8 % |
| | | F3 | 6/53 (11.3%) | 4.3 – 23.0 % | 8/52 (15.4%) | 6.9 – 28.1 % |
| | | F6 | 8/48 (16.7%) | 7.5 – 30.2 % | 9/53 (17.0%) | 8.1 – 29.8 % |

* All available cases were included in the calculations at all times (intention-to-treat analysis). B = baseline F3 = 3-mo follow-up F6 = 6-mo follow-up

**eTable 5. Comparison of the Change of Daily Sunscreen Use Between the Intervention and Control Groups**

| Pairwise Contrasts | | | | | | | | |
|---|---|---|---|---|---|---|---|---|
| Treatment group | Survey Wave pairwise Contrasts | Contrast Estimate | Std. Error | t | df | Adj. Sig. | 95% Confidence Interval | |
| | | | | | | | Lower | Upper |
| Control | 6-month follow-up - Baseline | -0.005 | 0.013 | -0.370 | 1432 | 0.711 | -0.030 | 0.020 |
| Intervention | 6-month follow-up - Baseline | 0.077 | 0.016 | 4.684 | 1181 | <0.001 | 0.045 | 0.109 |
| Difference in change between intervention and control | | 0.082 | 0.0205 | 3.986 | 2611 | <0.001 | 0.0415 | 0.122 |
| The sequential Šidák adjusted significance level is .05. | | | | | | | | |
| Confidence interval bounds are approximate. | | | | | | | | |

eTable 7 shows that the school grade had no significant influence on the use of the sunscreen. Therefore, this feature was removed from the model that was used for the pairwise contrasts related to the primary end point (eTables 5-6).

©2020 Brinker TJ et al. *JAMA Dermatology*.

**eTable 6. Gender-Specific Comparison of the Change of Daily Sunscreen Use Between the Intervention and Control Groups**

| Pairwise Contrasts | | | | | | | | |
|---|---|---|---|---|---|---|---|---|
| Gender & Treatment group | Survey Wave pairwise Contrasts | Contrast Estimate | Std. Error | t | df | Adj. Sig. | 95% Confidence Interval | |
| | | | | | | | Lower | Upper |
| Female Control | 6-month follow-up - Baseline | -0.003 | 0.022 | -0.118 | 1423 | 0.906 | -0.045 | 0.040 |
| Female Intervention | 6-month follow-up - Baseline | 0.125 | 0.025 | 4.913 | 1386 | <0.001 | 0.075 | 0.175 |
| Female | Change difference Intervention-Control | 0.128 | 0.335 | 3.816 | 2807 | <0.001 | 0.0621 | 0.193 |
| Male Control | 6-month follow-up - Baseline | -0.007 | 0.015 | -0.489 | 1445 | 0.625 | -0.037 | 0.022 |
| Male Intervention | 6-month follow-up - Baseline | 0.026 | 0.020 | 1.290 | 1487 | 0.197 | -0.014 | 0.066 |
| Male | Change difference Intervention-Control | 0.033 | 0.025 | 1.322 | 2930 | 0.186 | -0.0161 | 0.0827 |
| The sequential Šidák adjusted significance level is .05. | | | | | | | | |
| Confidence interval bounds are approximate. | | | | | | | | |

©2020 Brinker TJ et al. *JAMA Dermatology*.

## eTable 7. Analysis of the Influence of Different Factors on the Primary End Point

| Fixed Effects[a] | | | | |
|---|---|---|---|---|
| Source | F | df1 | df2 | Sig. |
| Corrected Model | 11.149 | 10 | 146 | <0.001 |
| Wave | 10.770 | 1 | 1483 | 0.001 |
| Interv_Control | 2.810 | 1 | 48 | 0.100 |
| Wave * Interv_Control | 14.422 | 1 | 1483 | <0.001 |
| Gender | 70.148 | 1 | 1558 | <0.001 |
| School grade | 0.554 | 3 | 48 | 0.648 |
| Fitzpatrick Skin Type | 3.664 | 3 | 1549 | 0.012 |
| Probability distribution: Binomial<br>Link function: Logit | | | | |
| a. Target: Daily sunscreen use | | | | |

©2020 Brinker TJ et al. *JAMA Dermatology*.

## Secondary end point: daily sunscreen use at 3 months follow-up

**eTable 8. Analysis of the Influence of Different Factors on the Secondary End Point "Daily Sunscreen Use at 3-mo Follow-up"**

| Fixed Effects[a] | | | | |
|---|---|---|---|---|
| Source | F | df1 | df2 | Sig. |
| Corrected Model | 11.925 | 10 | 139 | <0.001 |
| Wave | 5.320 | 1 | 1495 | 0.021 |
| Interv_Control | 3.535 | 1 | 46 | 0.066 |
| Wave * Interv_Control | 17.482 | 1 | 1495 | <0.001 |
| Gender | 80.211 | 1 | 1576 | <0.001 |
| School grade | 0.529 | 3 | 46 | 0.665 |
| Fitzpatrick Skin Type | 3.170 | 3 | 1556 | 0.023 |
| Probability distribution: Binomial<br>Link function: Logit | | | | |
| a. Target: Daily Sunscreen use | | | | |

eTable 8 shows that the school grade had no significant influence on the use of the sunscreen. Therefore, this feature was removed from the model that was used for the pairwise contrasts related to the secondary end point "daily sunscreen use at 3-month follow-up" (eTables 9-10).

**eTable 9. Comparison of the Change of Daily Sunscreen Use Between the Intervention and Control Groups**

| Pairwise Contrasts | | | | | | | | |
|---|---|---|---|---|---|---|---|---|
| Treatment group | Survey Wave pairwise Contrasts | Contrast Estimate | Std. Error | t | df | Adj. Sig. | 95% Confidence Interval | |
| | | | | | | | Lower | Upper |
| Control | 3-month follow-up - Baseline | -0.015 | 0.012 | -1.328 | 1475 | 0.184 | -0.038 | 0,007 |
| Intervention | 3-month follow-up - Baseline | 0.065 | 0.015 | 4.381 | 1171 | <0.001 | 0.036 | 0,094 |
| Difference in change between intervention and control | | 0.081 | 0.0186 | 4.338 | 2644 | <0.001 | 0.0442 | 0.117 |
| The sequential Šidák adjusted significance level is .05. | | | | | | | | |
| Confidence interval bounds are approximate. | | | | | | | | |

©2020 Brinker TJ et al. *JAMA Dermatology*.

**eTable 10. Gender-Specific Comparison of the Change of Daily Sunscreen Use Between the Intervention and Control Groups**

| Pairwise Contrasts | | | | | | | | |
|---|---|---|---|---|---|---|---|---|
| Gender & Treatment group | Survey Wave pairwise Contrasts | Contrast Estimate | Std. Error | t | df | Adj. Sig. | 95% Confidence Interval | |
| | | | | | | | Lower | Upper |
| Female Control | 3-month follow-up - Baseline | -0.004 | 0.021 | -0.209 | 1439 | 0.835 | -0.045 | 0.036 |
| Female Intervention | 3-month follow-up - Baseline | 0.120 | 0.024 | 4.998 | 1354 | <0.001 | 0.073 | 0.167 |
| Female | Change difference Intervention-Control | 0.124 | 0.032 | 3.941 | 2791 | <0.001 | 0.062 | 0.186 |
| Male Control | 3-month follow-up - Baseline | -0.028 | 0.013 | -2.147 | 1484 | 0.032 | -0.054 | -0.002 |
| Male Intervention | 3-month follow-up - Baseline | 0.009 | 0.018 | 0.488 | 1488 | 0.626 | -0.027 | 0.044 |
| Male | Change difference Intervention-Control | 0.037 | 0.022 | 1.660 | 2970 | 0.097 | -0.007 | 0.081 |
| The sequential Šidák adjusted significance level is .05. | | | | | | | | |
| Confidence interval bounds are approximate. | | | | | | | | |

©2020 Brinker TJ et al. *JAMA Dermatology*.

## Secondary end point: self-skin examinations

### eTable 11. Descriptive Characteristic of Pupils With at Least 1 Skin Self-examination in the Past 6 Months at Baseline and 3- and 6-Month Follow-up

| | | Time Point* | Intervention Group | 95%CI | Control Group | 95%CI |
|---|---|---|---|---|---|---|
| Total | | B | 184/734 (25.1%) | 22.0 – 28.4 % | 241/839 (28.7%) | 25.7 – 31.9 % |
| | | F3 | 255/618 (41.3%) | 37.3 – 45.3 % | 213/784 (27.2%) | 24.1 – 30.4 % |
| | | F6 | 300/607 (49.4%) | 45.4 – 53.5 % | 211/787 (26.8%) | 23.7 – 30.1 % |
| Gender | Male | B | 75/336 (22.3%) | 18.0 – 27.2 % | 104/425 (24.5%) | 20.5 – 28.8 % |
| | | F3 | 102/275 (37.1%) | 31.4 – 43.1 % | 92/395 (23.3%) | 19.2 – 27.8 % |
| | | F6 | 110/269 (40.9%) | 35.0 – 47.0 % | 87/392 (22.2%) | 18.2 – 26.6 % |
| | Female | B | 109/398 (27.4%) | 23.1 – 32.1 % | 137/414 (33.1%) | 28.6 – 37.9 % |
| | | F3 | 153/343 (44.6%) | 39.3 – 50.0 % | 121/389 (31.1%) | 26.5 – 36.0 % |
| | | F6 | 190/338 (56.2%) | 50.7 – 61.6 % | 124/395 (31.4%) | 26.8 – 36.2 % |
| School grade | 9th grade | B | 38/145 (26.2%) | 19.3 – 34.2 % | 40/113 (35.4%) | 26.8 – 45.0 % |
| | | F3 | 54/126 (42.9%) | 34.1 – 52.0 % | 34/108 (31.5%) | 22.9 – 41.1 % |
| | | F6 | 64/126 (50.8%) | 41.7 – 59.8 % | 31/108 (28.7%) | 20.4 – 38.2 % |
| | 10th grade | B | 54/221 (24.4%) | 18.9 – 30.6 % | 57/231 (24.7%) | 19.3 - 30.8 % |
| | | F3 | 70/181 (38.7%) | 31.5 – 46.2 % | 54/218 (24.8%) | 19.2 – 31.1 % |
| | | F6 | 90/189 (47.6%) | 40.3 – 55.0 % | 48/213 (22.5%) | 17.1 – 28.7 % |
| | 11th grade | B | 43/178 (24.2%) | 18.1 – 31.1 % | 71/246 (28.9%) | 23.3 – 35.0 % |
| | | F3 | 54/149 (36.2%) | 28.5 – 44.5 % | 60/231 (26.0%) | 20.4 – 32.1 % |
| | | F6 | 68/147 (46.3%) | 38.0 – 54.7 % | 65/237 (27.4%) | 21.8 – 33.6 % |
| | 12th grade | B | 49/190 (25.8%) | 19.7 – 32.6 % | 73/249 (29.3%) | 23.7 – 35.4 % |
| | | F3 | 77/162 (47.5%) | 39.6 – 55.5 % | 65/227 (28.6%) | 22.8 – 35.0 % |
| | | F6 | 78/145 (53.8%) | 45.3 – 62.1 % | 67/229 (29.3%) | 23.5 – 35.6 % |
| Fitzpatrick Skin Type | Type I or II | B | 14/46 (30.4%) | 17.7 – 45.8 % | 20/70 (28.6%) | 18.4 – 40.6 % |
| | | F3 | 20/39 (51.3%) | 34.8 – 67.6 % | 16/64 (25.0%) | 15.0 – 37.4 % |
| | | F6 | 20/37 (54.1%) | 36.9 – 70.5 % | 17/65 (26.2%) | 16.0 – 38.5 % |
| | Type III | B | 68/256 (26.6%) | 21.3 – 32.4 % | 103/293 (35.2%) | 29.7 – 40.9 % |
| | | F3 | 91/210 (43.3%) | 36.5 – 50.3 % | 90/275 (32.7%) | 27.2 – 38.6 % |
| | | F6 | 102/211 (48.3%) | 41.4 – 55.3 % | 86/273 (31.5%) | 26.0 – 37.4 % |
| | Type IV | B | 87/368 (23.6%) | 19.4 – 28.3 % | 103/421 (24.5%) | 20.4 – 28.9 % |
| | | F3 | 127/316 (40.2%) | 34.7 – 45.8 % | 96/393 (24.4%) | 20.3 – 29.0 % |
| | | F6 | 159/311 (51.1%) | 45.4 – 56.8 % | 97/396 (24.5%) | 20.3 – 29.0 % |
| | Type V | B | 15/64 (23.4%) | 13.8 – 35.7 % | 15/55 (27.3%) | 16.1 – 41.0 % |
| | | F3 | 17/53 (32.1%) | 19.9 – 46.3 % | 11/52 (21.2%) | 11.1 – 34.7 % |
| | | F6 | 19/48 (39.6%) | 25.8 – 54.7 % | 11/53 (20.8%) | 10.8 – 34.1 % |

* Because the dropouts do not distort the secondary objectives, all available cases were included in the calculations at all times (intention-to-treat analysis).
95% confidence intervals were calculated using the Clopper and Pearson method.
B = baseline 3F= 3-mo follow-up 6F = 6-mo follow-up

©2020 Brinker TJ et al. *JAMA Dermatology*.

**eTable 12. Comparison of the Change of the Prevalence of Pupils With at Least 1 Skin Self-examination Between the Intervention and Control Groups**

| Pairwise Contrasts | | | | | | | | |
|---|---|---|---|---|---|---|---|---|
| Treatment group | Survey Wave pairwise Contrasts | Contrast Estimate | Std. Error | t | df | Adj. Sig. | 95% Confidence Interval | |
| | | | | | | | Lower | Upper |
| Control | 6-month follow-up - Baseline | -0.020 | 0.017 | -1.209 | 1449 | 0.227 | -0.054 | 0,013 |
| Intervention | 6-month follow-up - Baseline | 0.243 | 0.021 | 11.571 | 1266 | <0.001 | 0.202 | 0,284 |
| Difference in change between intervention and control | | 0.264 | 0.027 | 9.869 | 2713 | <0.001 | 0.211 | 0.316 |
| The sequential Šidák adjusted significance level is .05. | | | | | | | | |
| Confidence interval bounds are approximate. | | | | | | | | |

eTable 14 shows that the school grade had no significant influence on self-skin examination. Therefore, this feature was removed from the model that was used for the pairwise contrasts related to the secondary end point “skin self-examinations” (eTables 12-13). The Fitzpatrick skin type does not have a significant effect (see eTable 14), but was left in the model for adjustment.

©2020 Brinker TJ et al. *JAMA Dermatology*.

**eTable 13. Gender-Specific Comparison of the Change of the Prevalence of Pupils With at Least 1 Skin Self-examination Between the Intervention and Control Groups**

| Pairwise Contrasts | | | | | | | | |
|---|---|---|---|---|---|---|---|---|
| Gender & Treatment group | Survey Wave pairwise Contrasts | Contrast Estimate | Std. Error | t | df | Adj. Sig. | 95% Confidence Interval | |
| | | | | | | | Lower | Upper |
| Female Control | 6-month follow-up - Baseline | -0.017 | 0.025 | -0.693 | 1434 | 0.488 | -0.066 | 0.031 |
| Female Intervention | 6-month follow-up - Baseline | 0.282 | 0.028 | 10.178 | 1476 | <0.001 | 0.228 | 0.337 |
| Female | Change difference Intervention-Control | 0.300 | 0.037 | 8.037 | 2908 | <0.001 | 0.227 | 0.373 |
| Male Control | 6-month follow-up - Baseline | -0.023 | 0.023 | -1.036 | 1460 | 0.300 | -0.068 | 0.021 |
| Male Intervention | 6-month follow-up - Baseline | 0.192 | 0.030 | 6.354 | 1455 | <0.001 | 0.132 | 0.251 |
| Male | Change difference Intervention-Control | 0.215 | 0.038 | 5.710 | 2913 | <0.001 | 0.141 | 0.289 |
| The sequential Šidák adjusted significance level is .05. | | | | | | | | |
| Confidence interval bounds are approximate. | | | | | | | | |

**eTable 14. Analysis of the Influence of Different Factors on the Secondary End Point "Skin Self-examination Within the Past 6 Months"**

| Fixed Effects[a] | | | | |
|---|---|---|---|---|
| Source | F | df1 | df2 | Sig. |
| Corrected Model | 17.307 | 10 | 128 | <0.001 |
| Wave | 59.725 | 1 | 1496 | <0.001 |
| Interv_Control | 9.739 | 1 | 46 | 0.003 |
| Wave * Interv_Control | 87.814 | 1 | 1496 | <0.001 |
| Gender | 18.260 | 1 | 1561 | <0.001 |
| School grade | 0.823 | 3 | 46 | 0.488 |
| Fitzpatrick Skin Type | 1.846 | 3 | 1560 | 0.137 |
| Probability distribution: Binomial<br>Link function: Logit | | | | |
| a. Target: At least one self-skin examination in the last 6 months | | | | |

©2020 Brinker TJ et al. *JAMA Dermatology*.

## Secondary end point: tanning sessions

### eTable 15. Descriptive Characteristic of Pupils With at Least 1 Tanning Session in the Past 30 Days at Baseline and 3- and 6-Month Follow-up

| | | Time Point* | Intervention Group | 95%CI | Control Group | 95%CI |
|---|---|---|---|---|---|---|
| Total | | B | 138/734 (18.8%) | 16.0 – 21.8 % | 109/839 (13.0%) | 10.8 – 15.5 % |
| | | F3 | 74/618 (12.0%) | 9.5 – 14.8 % | 90/784 (11.5%) | 9.3 – 13.9 % |
| | | F6 | 92/607 (15.2%) | 12.4 – 18.3 % | 107/787 (13.6%) | 11.3 – 16.2 % |
| Gender | Male | B | 41/336 (12.2%) | 8.9 – 16.2 % | 35/425 (8.2%) | 5.8 – 11.3 % |
| | | F3 | 28/275 (10.2%) | 6.9 – 14.4 % | 22/395 (5.6%) | 3.5 – 8.3 % |
| | | F6 | 24/269 (8.9%) | 5.8 – 13.0 % | 21/392 (5.4%) | 3.3 – 8.1 % |
| | Female | B | 97/398 (24.4%) | 20.2 – 28.9 % | 74/414 (17.9%) | 14.3 – 21.9 % |
| | | F3 | 46/343 (13.4%) | 10.0 – 17.5 % | 68/389 (17.5%) | 13.8 – 21.6 % |
| | | F6 | 68/338 (20.1%) | 16.0 – 24.8 % | 86/395 (21.8%) | 17.8 – 26.2 % |
| School grade | 9th grade | B | 35/145 (24.1%) | 17.4 – 31.9 % | 17/113 (15.0%) | 9.0 – 23.0 % |
| | | F3 | 14/126 (11.1%) | 6.2 – 17.9 % | 21/108 (19.4%) | 12.5 – 28.2 % |
| | | F6 | 18/126 (14.3%) | 8.7 – 21.6 % | 22/108 (20.4%) | 13.2 – 29.2 % |
| | 10th grade | B | 30/221 (13.6%) | 9.3 – 18.8 % | 37/231 (16.0%) | 11.5 – 21.4 % |
| | | F3 | 23/181 (12.7%) | 8.2 – 18.5 % | 26/218 (11.9%) | 7.9 – 17.0 % |
| | | F6 | 26/189 (13.8%) | 9.2 – 19.5 % | 31/213 (14.6%) | 10.1 – 20.0 |
| | 11th grade | B | 39/178 (21.9%) | 16.1 – 28.7 % | 28/246 (11.4%) | 7.7 – 16.0 % |
| | | F3 | 16/149 (10.7%) | 6.3 – 16.9 % | 24/231 (10.4%) | 6.8 – 15.1 % |
| | | F6 | 28/147 (19.0%) | 13.0 – 26.3 % | 26/237 (11.0%) | 7.3 – 15.7 % |
| | 12th grade | B | 34/190 (17.9%) | 12.7 – 24.1 % | 27/249 (10.8%) | 7.3 – 15.4 % |
| | | F3 | 21/162 (13.0%) | 8.2 – 19.1 % | 19/227 (8.4%) | 5.1 – 12.8 % |
| | | F6 | 20/145 (13.8%) | 8.6 – 20.5 % | 28/229 (12.2%) | 8.3 – 17.2 % |
| Fitzpatrick Skin Type | Type I or II | B | 8/46 (17.4%) | 7.8 – 31.4 % | 9/70 (12.9%) | 6.1 – 23.0 % |
| | | F3 | 2/39 (5.1%) | 0.6 – 17.3 % | 11/64 (17.2%) | 8.9 – 28.7 % |
| | | F6 | 4/37 (10.8%) | 3.0 – 25.4 % | 10/65 (15.4%) | 7.6 – 26.5 % |
| | Type III | B | 56/256 (21.9%) | 17.0 – 27.4 % | 41/293 (14.0%) | 10.2 – 18.5 % |
| | | F3 | 26/210 (12.4%) | 8.2 – 17.6 % | 32/275 (11.6%) | 8.1 – 16.0 % |
| | | F6 | 36/211 (17.1%) | 12.2 – 22.8 % | 40/273 (14.7%) | 10.7 – 19.4 % |
| | Type IV | B | 64/368 (17.4%) | 13.7 – 21.7 % | 49/421 (11.6%) | 8.7 – 15.1 % |
| | | F3 | 37/316 (11.7%) | 8.4 – 15.8 % | 41/393 (10.4%) | 7.6 – 13.9 % |
| | | F6 | 41/311 (13.2%) | 9.6 – 17.5 % | 51/396 (12.9%) | 9.7 – 16.6 % |
| | Type V | B | 10/64 (15.6%) | 7.8 – 26.9 % | 10/55 (18.2%) | 9.1 – 30.9 % |
| | | F3 | 9/53 (17.0%) | 8.1 – 29.8 % | 6/52 (11.5%) | 4.4 – 23.4 % |
| | | F6 | 11/48 (22.9%) | 12.0 – 37.3 % | 6/53 (11.3%) | 4.3 – 23.0 % |

* Because the drop-outs do not distort the secondary objectives, all available cases were included in the calculations at all times (intention-to-treat analysis).
95% confidence intervals were calculated using the Clopper and Pearson method.
B = baseline 3F = 3-mo follow-up 6F = 6-mo follow-up

©2020 Brinker TJ et al. *JAMA Dermatology*.

**eTable 16. Comparison of the Change of the Prevalence of Pupils With at Least 1 Tanning Session in the Past 30 Days Between the Intervention and Control Groups**

| Pairwise Contrasts without distinction by gender | | | | | | | | |
|---|---|---|---|---|---|---|---|---|
| Treatment group | Survey Wave pairwise Contrasts | Contrast Estimate | Std. Error | t | df | Adj. Sig. | 95% Confidence Interval | |
| | | | | | | | Lower | Upper |
| Control | 6-month follow-up - Baseline | 0.006 | 0.013 | 0.431 | 1456 | 0.667 | -0.020 | 0.031 |
| Intervention | 6-month follow-up - Baseline | **-0.035** | 0.015 | -2.341 | 1559 | **0.019** | -0.065 | -0.006 |
| Difference in change F6-B between intervention and control | | **-0.041** | 0.020 | -2.053 | 3013 | **0.040** | -0.080 | -0.002 |
| Control | 3-month follow-up - Baseline | -0.016 | 0.012 | -1.299 | 1501 | 0.350 | -0.044 | 0.012 |
| Intervention | 3-month follow-up - Baseline | **-0.062** | 0.015 | -4.153 | 1141 | **0.000** | -0.098 | -0.027 |
| Difference in change F3-B between intervention and control | | **-0.046** | 0.019 | -2.423 | 2640 | **0.015** | -0.083 | -0.009 |
| Control | 6-month follow-up - 3-month follow-up | 0.022 | 0.008 | 2.653 | 1337 | 0.024 | 0.002 | 0.041 |
| Intervention | 6-month follow-up - 3-month follow-up | **0.027** | 0.009 | 2.982 | 1281 | **0.006** | 0.007 | 0.048 |
| Difference in change F6-F3 between intervention and control | | 0.005 | 0.012 | 0.416 | 2616 | 0.677 | -0.019 | 0.029 |
| The sequential Šidák adjusted significance level is .05. | | | | | | | | |
| Confidence interval bounds are approximate. B = baseline. F3 = 3 months follow-up. F6 = 6 months follow-up. | | | | | | | | |

eTable 18 shows that the school grade and Fitzpatrick skin type had no significant influence on the tanning sessions. Therefore, these two features were removed from the model that was used for the pairwise contrasts related to the secondary end point "tanning sessions" (eTables 16-17).

**eTable 17. Gender-Specific Comparison of the Change of the Prevalence of Pupils With at Least 1 Tanning Session in the Past 30 Days Between the Intervention and Control Groups**

| Pairwise Contrasts with Gender and Treatment Group combined | | | | | | | | |
|---|---|---|---|---|---|---|---|---|
| Gender & Treatment group | Survey Wave pairwise Contrasts | Contrast Estimate | Std. Error | t | df | Adj. Sig. | 95% Confidence Interval | |
| | | | | | | | Lower | Upper |
| Female Control | 6-month follow-up - Baseline | 0.036 | 0.023 | 1.596 | 1453 | 0.209 | -0.015 | 0.087 |
| Female Intervention | 6-month follow-up - Baseline | -0.044 | 0.024 | -1.810 | 1537 | 0.070 | -0.092 | 0.004 |
| Female F6-B | Change difference Intervention-Control | -0.080 | 0.033 | -2.402 | 2988 | 0.016 | -0.145 | -0.015 |
| Female Control | 3-month follow-up - Baseline | -0.005 | 0.022 | -0.248 | 1471 | 0.804 | -0.048 | 0.037 |

©2020 Brinker TJ et al. *JAMA Dermatology*.

| | | | | | | | | |
|---|---|---|---|---|---|---|---|---|
| Female Intervention | 3-month follow-up - Baseline | -0.109 | 0.023 | -4.714 | 1144 | 0.000 | -0.164 | -0.054 |
| Female F3-B | Change difference Intervention-Control | -0.104 | 0.032 | -3.235 | 2613 | 0.001 | -0.167 | -0.041 |
| Female Control | 6-month follow-up - 3-month follow-up | 0.042 | 0.015 | 2.861 | 1337 | 0.013 | 0.007 | 0.076 |
| Female Intervention | 6-month follow-up - 3-month follow-up | 0.065 | 0.015 | 4.343 | 1019 | 0.000 | 0.031 | 0.098 |
| Female F6-F3 | Change difference Intervention-Control | 0.023 | 0.022 | 1.065 | 2354 | 0.287 | -0.019 | 0.065 |
| Male Control | 6-month follow-up - Baseline | -0.023 | 0.014 | -1.690 | 1504 | 0.174 | -0.054 | 0.008 |
| Male Intervention | 6-month follow-up - Baseline | -0.031 | 0.020 | -1.571 | 1580 | 0.310 | -0.077 | 0.016 |
| Male F6-B | Change difference Intervention-Control | -0.008 | 0.025 | -0.325 | 3082 | 0.745 | -0.056 | 0.040 |
| Male Control | 3-month follow-up - Baseline | -0.026 | 0.013 | -1.968 | 1506 | 0.140 | -0.059 | 0.006 |
| Male Intervention | 3-month follow-up - Baseline | -0.016 | 0.020 | -0.811 | 1556 | 0.443 | -0.056 | 0.024 |
| Male F3-B | Change difference Intervention-Control | 0.010 | 0.024 | 0.416 | 3060 | 0.677 | -0.037 | 0.057 |
| Male Control | 6-month follow-up - 3-month follow-up | 0.003 | 0.008 | 0.421 | 1341 | 0.674 | -0.012 | 0.019 |
| Male Intervention | 6-month follow-up - 3-month follow-up | -0.015 | 0.013 | -1.142 | 1390 | 0.443 | -0.043 | 0.014 |
| Male F6-F3 | Change difference Intervention-Control | -0.018 | 0.015 | -1.169 | 2729 | 0.242 | -0.048 | 0.012 |

The sequential Šidák adjusted significance level is .05.

Confidence interval bounds are approximate. B = baseline. F3 = 3 months follow-up. F6 = 6 months follow-up.

©2020 Brinker TJ et al. *JAMA Dermatology*.

**eTable 18. Analysis of the Influence of Different Factors on the Secondary End Point "at Least 1 Tanning Session Within the Past 30 Days"**

| Fixed Effects[a] | | | | |
|---|---|---|---|---|
| Source | F | df1 | df2 | Sig. |
| Corrected Model | 8.399 | 10 | 138 | 0.000 |
| Wave | 1.915 | 1 | 1470 | 0.167 |
| Interv_Control | 1.201 | 1 | 49 | 0.279 |
| Wave * Interv_Control | 4.051 | 1 | 1470 | 0.044 |
| Gender | 69.938 | 1 | 1554 | 0.000 |
| School grade | 0.913 | 3 | 48 | 0.442 |
| Fitzpatrick Skin Type | 1.469 | 3 | 1538 | 0.221 |
| Probability distribution: Binomial<br>Link function: Logit | | | | |
| a. Target: At least one tanning session within the last 30 days | | | | |

©2020 Brinker TJ et al. *JAMA Dermatology*.

## Attrition Analysis

### eTable 19. Dropouts in Relation to Assigned Group

**a) Crosstab**

| | | | Lost to follow-up at 6 months follow-up | | |
|---|---|---|---|---|---|
| | | | No dropout | Dropout | Total |
| Group (control or intervention) | Control | Count | 787 | 52 | 839 |
| | | Row % | 93,8% | 6,2% | 100,0% |
| | Intervention | Count | 607 | 127 | 734 |
| | | Row % | 82,7% | 17,3% | 100,0% |
| Total | | Count | 1394 | 179 | 1573 |
| | | Row % | 88,6% | 11,4% | 100,0% |

**b) Chi-Square Tests**

| | Value | df | Asymp. Sig. (2-sided) | Exact Sig. (2-sided) | Exact Sig. (1-sided) |
|---|---|---|---|---|---|
| Pearson Chi-Square | 47,871[a] | 1 | ,000 | | |
| Continuity Correction[b] | 46,777 | 1 | ,000 | | |
| Likelihood Ratio | 48,709 | 1 | ,000 | | |
| Fisher's Exact Test | | | | **,000** | ,000 |
| Linear-by-Linear Association | 47,841 | 1 | ,000 | | |
| N of Valid Cases | 1573 | | | | |

a. 0 cells (0,0%) have expected count less than 5. The minimum expected count is 83,53.

b. Computed only for a 2x2 table

©2020 Brinker TJ et al. *JAMA Dermatology*.

**eTable 20. Dropouts in Relation to Sunscreen Use**

**a) Crosstab**

| | | | Lost to follow-up at 6 months follow-up | | |
|---|---|---|---|---|---|
| | | | No dropout | Dropout | Total |
| Daily sunscreen use | No | Count | 1185 | 153 | 1338 |
| | | Row % | 88,6% | 11,4% | 100,0% |
| | Yes | Count | 209 | 26 | 235 |
| | | Row % | 88,9% | 11,1% | 100,0% |
| Total | | Count | 1394 | 179 | 1573 |
| | | Row % | 88,6% | 11,4% | 100,0% |

**b) Chi-Square Tests**

| | Value | df | Asymp. Sig. (2-sided) | Exact Sig. (2-sided) | Exact Sig. (1-sided) |
|---|---|---|---|---|---|
| Pearson Chi-Square | ,027[a] | 1 | ,869 | | |
| Continuity Correction[b] | ,003 | 1 | ,957 | | |
| Likelihood Ratio | ,027 | 1 | ,868 | | |
| Fisher's Exact Test | | | | **1,000** | ,487 |
| Linear-by-Linear Association | ,027 | 1 | ,869 | | |
| N of Valid Cases | 1573 | | | | |

a. 0 cells (0,0%) have expected count less than 5. The minimum expected count is 26,74.

b. Computed only for a 2x2 table

©2020 Brinker TJ et al. *JAMA Dermatology*.

**eTable 21. Dropouts in Relation to Skin Self-examinations**

**a) Crosstab**

| | | | Lost to follow-up at 6 months follow-up | | |
|---|---|---|---|---|---|
| | | | No dropout | Dropout | Total |
| Self-skin examination in the previous 6 months | None | Count | 1013 | 135 | 1148 |
| | | Row % | 88,2% | 11,8% | 100,0% |
| | At least once | Count | 381 | 44 | 425 |
| | | Row % | 89,6% | 10,4% | 100,0% |
| Total | | Count | 1394 | 179 | 1573 |
| | | Row % | 88,6% | 11,4% | 100,0% |

**b)Chi-Square Tests**

| | Value | df | Asymp. Sig. (2-sided) | Exact Sig. (2-sided) | Exact Sig. (1-sided) |
|---|---|---|---|---|---|
| Pearson Chi-Square | ,609[a] | 1 | ,435 | | |
| Continuity Correction[b] | ,477 | 1 | ,490 | | |
| Likelihood Ratio | ,619 | 1 | ,431 | | |
| Fisher's Exact Test | | | | **,475** | ,247 |
| Linear-by-Linear Association | ,608 | 1 | ,435 | | |
| N of Valid Cases | 1573 | | | | |

a. 0 cells (0,0%) have expected count less than 5. The minimum expected count is 48,36.
b. Computed only for a 2x2 table

©2020 Brinker TJ et al. *JAMA Dermatology*.

**eTable 22. Dropouts in Relation to Tanning Sessions**

### a) Crosstab

| | | | Lost to follow-up at 6 months follow-up | | |
|---|---|---|---|---|---|
| | | | No dropout | Dropout | Total |
| Tanning sessions in the past 30 days | No session | Count | 1178 | 148 | 1326 |
| | | Row % | 88,8% | 11,2% | 100,0% |
| | At least one session | Count | 216 | 31 | 247 |
| | | Row % | 87,4% | 12,6% | 100,0% |
| Total | | Count | 1394 | 179 | 1573 |
| | | Row % | 88,6% | 11,4% | 100,0% |

### b) Chi-Square Tests

| | Value | df | Asymp. Sig. (2-sided) | Exact Sig. (2-sided) | Exact Sig. (1-sided) |
|---|---|---|---|---|---|
| Pearson Chi-Square | ,398[a] | 1 | ,528 | | |
| Continuity Correction[b] | ,273 | 1 | ,602 | | |
| Likelihood Ratio | ,389 | 1 | ,533 | | |
| Fisher's Exact Test | | | | **,514** | ,296 |
| Linear-by-Linear Association | ,398 | 1 | ,528 | | |
| N of Valid Cases | 1573 | | | | |

a. 0 cells (0,0%) have expected count less than 5. The minimum expected count is 28,11.

b. Computed only for a 2x2 table

**eTable 23. Dropouts in Relation to Gender**

**a) Crosstab**

| | | | Lost to follow-up (F6) | | |
|---|---|---|---|---|---|
| | | | 0 kein Dropout | 1 Dropout | Total |
| Gender | 0 male | Count | 661 | 100 | 761 |
| | | Row % | 86,9% | 13,1% | 100,0% |
| | 1 female | Count | 733 | 79 | 812 |
| | | Row % | 90,3% | 9,7% | 100,0% |
| Total | | Count | 1394 | 179 | 1573 |
| | | Row % | 88,6% | 11,4% | 100,0% |

**b) Chi-Square Tests**

| | Value | df | Asymp. Sig. (2-sided) | Exact Sig. (2-sided) | Exact Sig. (1-sided) |
|---|---|---|---|---|---|
| Pearson Chi-Square | 4,534[a] | 1 | ,033 | | |
| Continuity Correction[b] | 4,202 | 1 | ,040 | | |
| Likelihood Ratio | 4,536 | 1 | ,033 | | |
| Fisher's Exact Test | | | | **,039** | ,020 |
| Linear-by-Linear Association | 4,531 | 1 | ,033 | | |
| N of Valid Cases | 1573 | | | | |

a. 0 cells (0,0%) have expected count less than 5. The minimum expected count is 86,60.

b. Computed only for a 2x2 table

©2020 Brinker TJ et al. *JAMA Dermatology*.
